# Supplementary material for: Cellular Fitness Phenotypes of Cancer Target Genes from Oncobiology to Cancer Therapeutics
Source: Cells. 2021 Feb 18;10(2):433. doi: 10.3390/cells10020433 (PMC7921985; doi:10.3390/cells10020433)
Supplement: Supplementary file 1 [file cells-10-00433-s001.pdf]

SUPPLEMENTARY FILE

Cellular FitnessPhenotype of Cancer Target Genes in Cancer Therapeutics

Bijesh George, P. Mukundan Pillai, Aswathy Mary Paul, Revikumar Amjesh, Kim Leitzel,

Suhail M. Ali, Oleta Sandiford, Allan Lipton, Pranela Rameshwar, Gabriel N. Hortobagyi,

Madhavan Radhakrishna Pillai, and Rakesh Kumar

Supplementary Figures

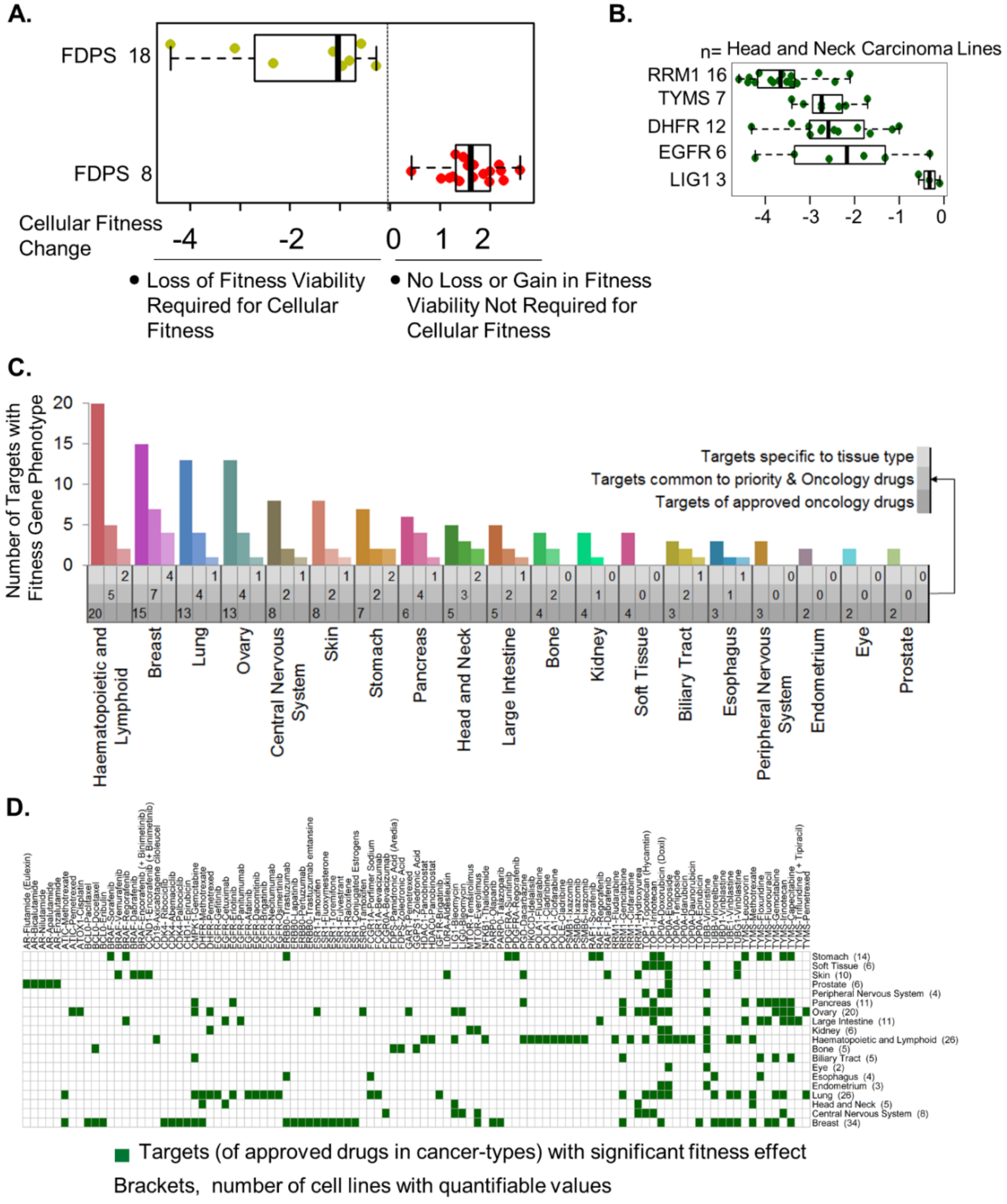

Supplementary Figure S1: Fitness-dependency of cellular targets of approved oncology drugs in cancer cell lines. **A**, Distribution of alterations in the cellular fitness in esophageal cancer cell lines upon selective knockdown of FSPS test gene. The values are plotted as boxplots with positive and negative changes in the cellular fitness for each cell line, represented by a single dot. **B,** Effect of knocking down of indicated targets in Head and Neck carcinoma cell lines. **C,** Distribution of 47 cancer targets of FDA-approved drugs, including, a subset of its 15 priority therapeutic targets in across cancer-types for which drugs targeting these cellular targets are approved. **D**, Distribution of 47 fitness targets across 19 cancer types for which drugs targeting these molecules are approved corresponds to the loss of Fitness score for were taken from Cancer Dependency Map (23).

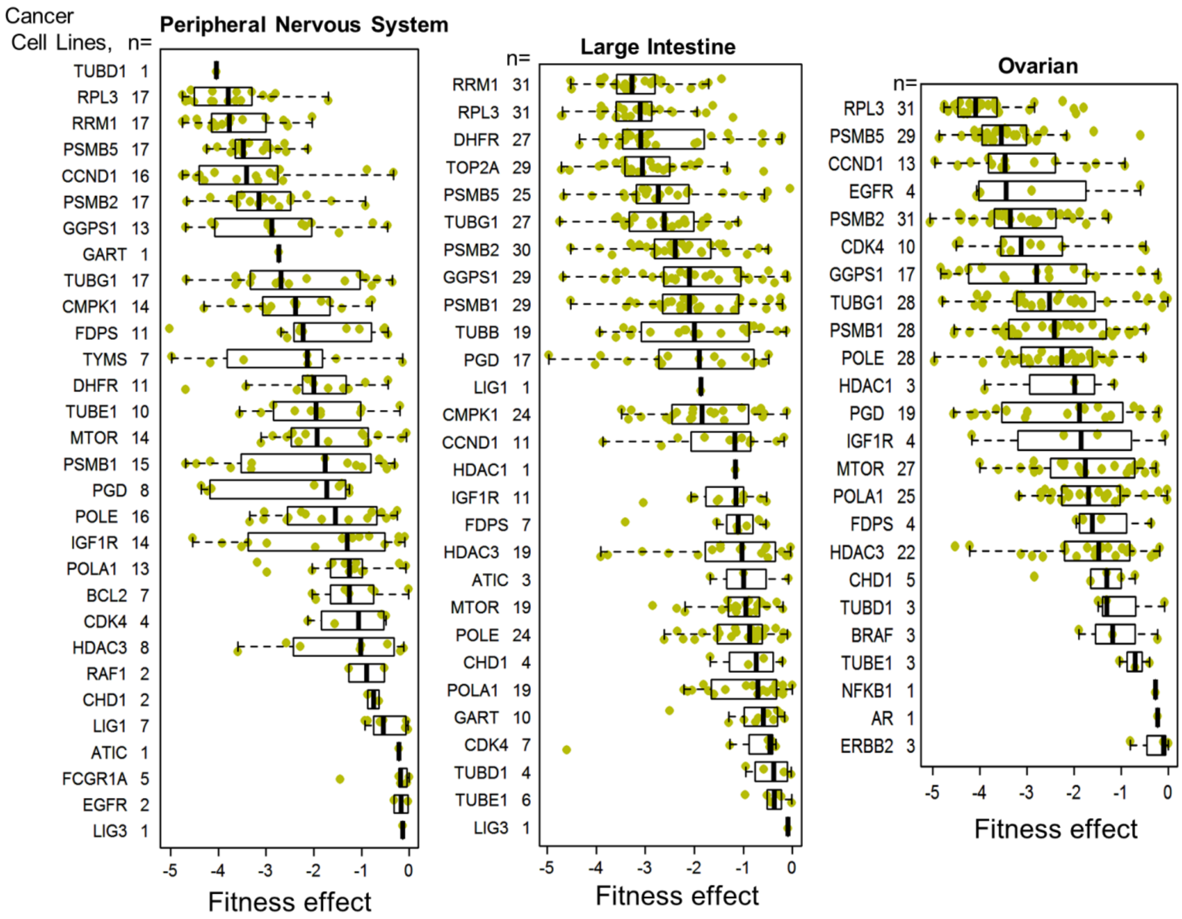

Supplementary Figure S2: Cellular targets of approved oncology drugs as excellent fitness genes in cancer-types for which drugs targeting these are not approved. Distribution of 43 cellular fitness genes with a significant loss of cellular fitness upon their depletion across peripheral nervous system, large intestine and ovarian cell lines for which drugs targeting these molecules are not approved.

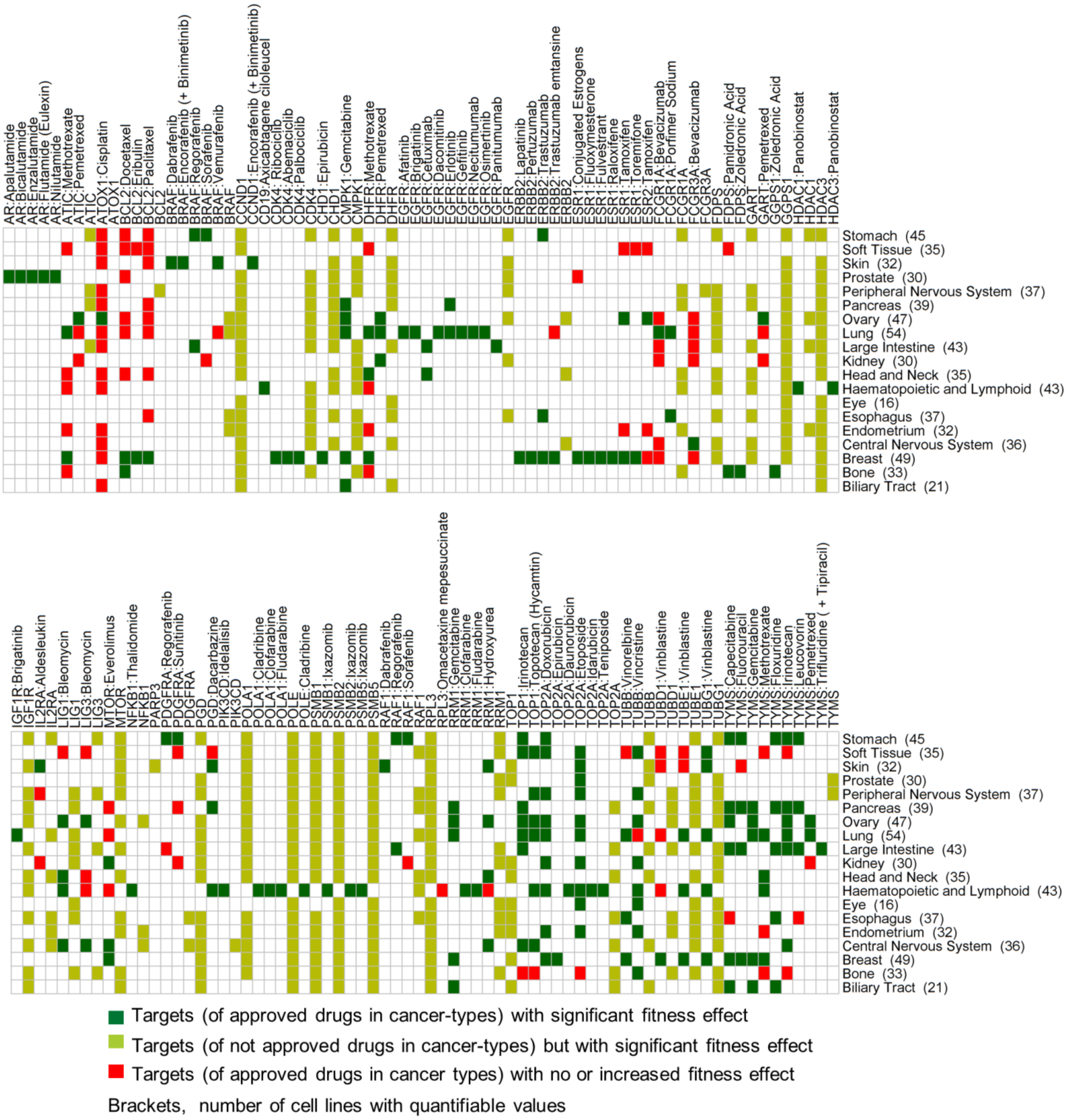

Supplementary Figure S3: Status of fitness-dependency of 47 oncology drug targets in cancer-dependency map. Overall distribution of cellular targets with excellent fitness-dependency effect (upon knocking down of these targets) in cancer-types for which drugs targeting these molecules are approved (dark green, targets n=47) and not approved (light green, targets n= 43), and distribution of cellular

targets (of approved drugs) with a positive fitness effect upon knocking down of these molecules (red, targets n=35).

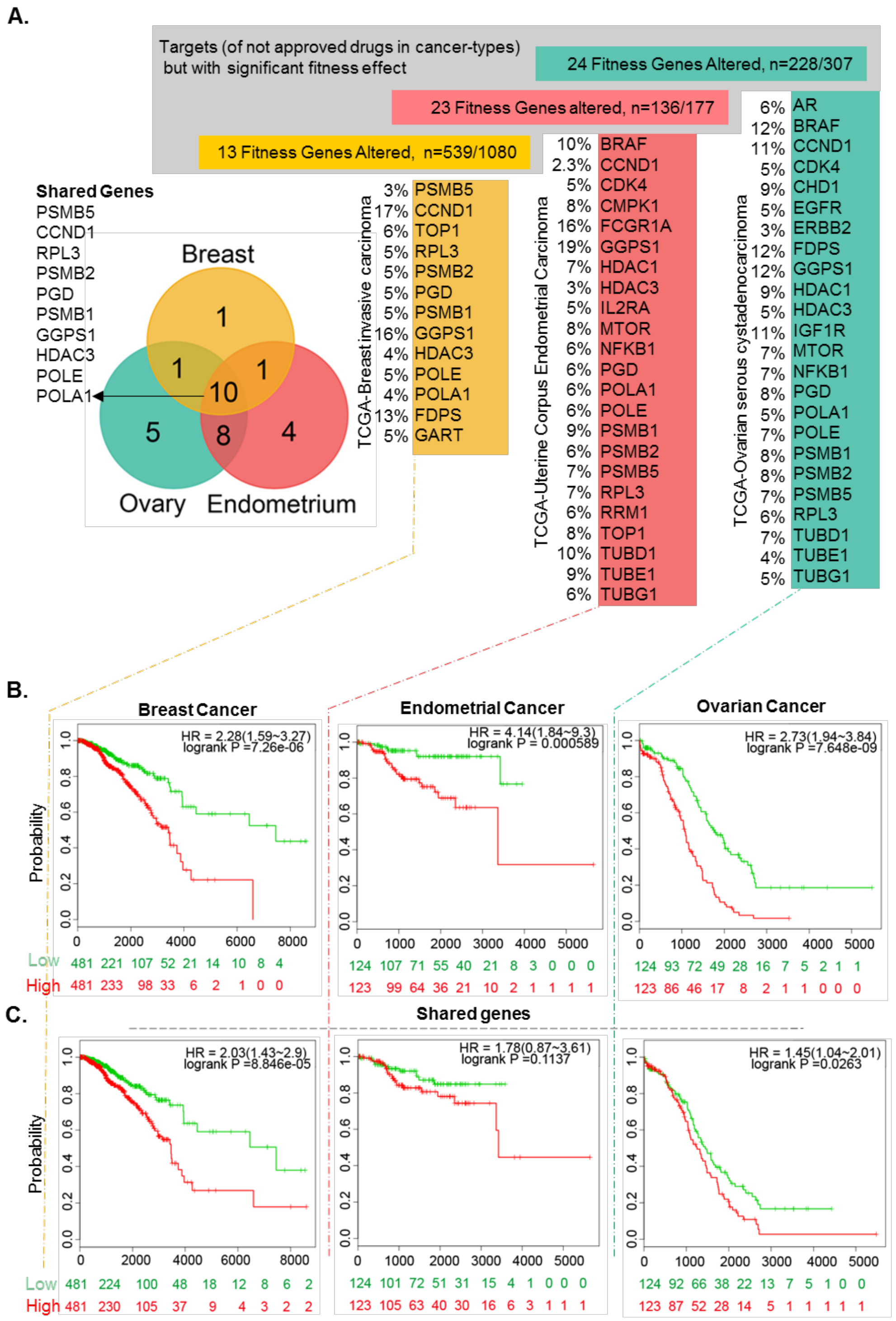

Supplementary Figure 4

Supplementary Figure S4: Widespread overexpression and significance of cellular fitness targets in women's cancer. A, Overexpression of cellular fitness targets in breast, ovarian and endometrial cancers in TGCA datasets. Venn Diagram of genes shared between Breast, Ovarian and endometrial cancer [left panel].for each cancer the rate of alteration of gene list has been listed next the the gene symbol, the alteration data was taken from cBioPortal (29, 30).**B & C**, Multi-variant survival analysis of 13 fitness genes in breast cancer, 24 fitness genes in ovarian cancer and 23 fitness genes in endometrial cancer (b), and shared 10 fitness genes in three cancers (c). Survival plot using SurvExpress (32) has plotted with the list given above for each corresponding cancer. TCGA Breast invasive carcinoma, TCGA - Ovarian serous cystadenocarcinoma and TCGA - Uterine Corpus Endometrial Carcinoma using data from cBioPortal (29, 30).

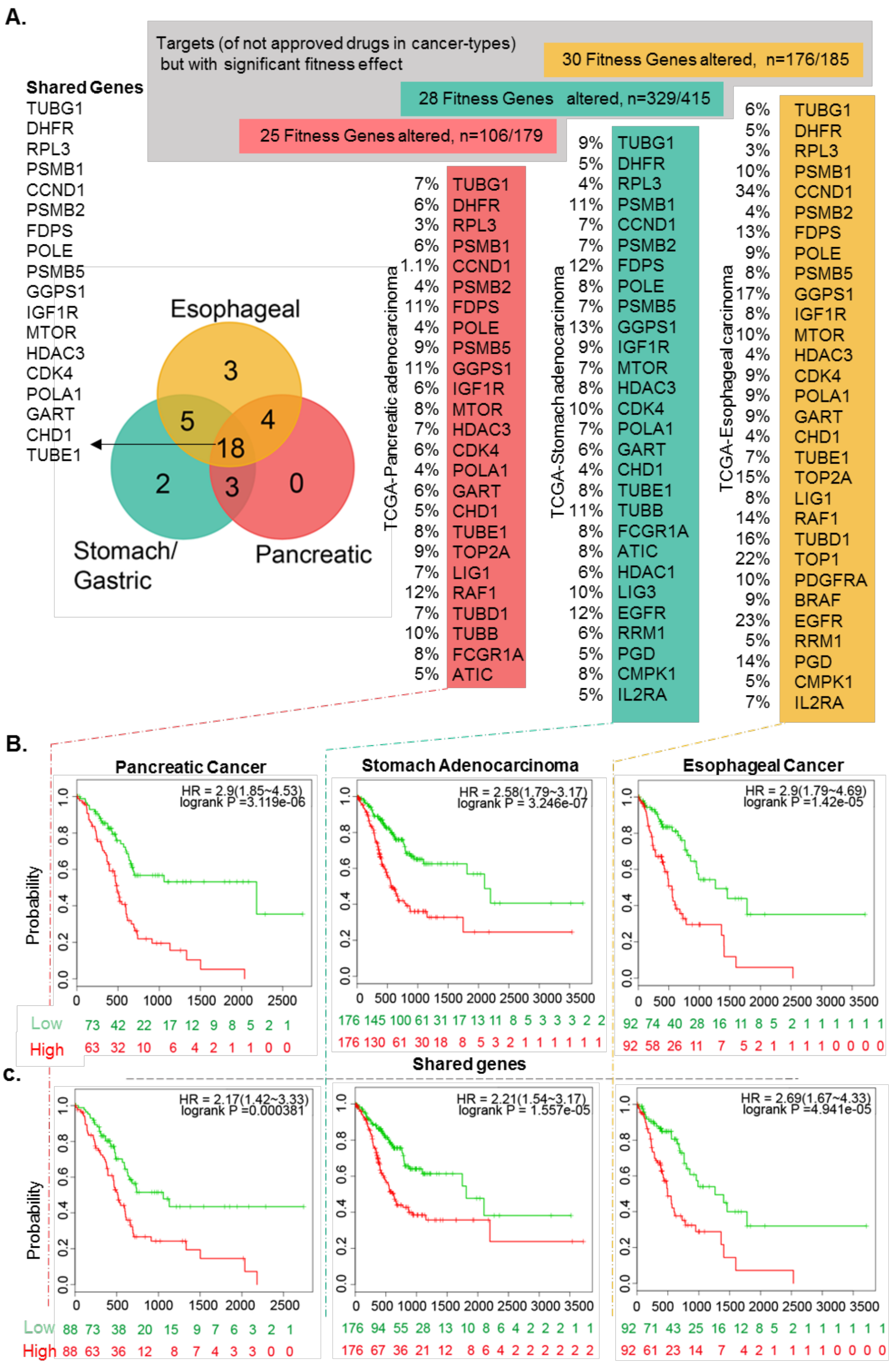

Supplementary Figure 5

Supplementary Figure S5: Widespread overexpression and significance of cellular fitness targets in digestive cancers. A, Overexpression of cellular fitness targets in pancreatic, stomach and esophageal cancers in TGCA datasets. Venn Diagram of genes shared between esophageal, Pancreatic and Stomach cancer [left panel for each cancer the rate of alteration of gene list has been listed next the gene symbol, the alteration data was taken from cBioPortal (29, 30).**B & C**, Multi-variant survival analysis of 25 fitness genes in pancreatic cancer, 28 fitness genes in stomach cancer and 30 fitness genes in esophageal cancer (b), and shared 18 fitness genes in three cancers (c). Survival plot using SurvExpress (32) has plotted with the list given above for each corresponding cancer (TCGA esophageal carcinoma, TCGA - Pancreatic adenocarcinoma and TCGA - Stomach adenocarcinoma) from cBioPortal (29, 30).

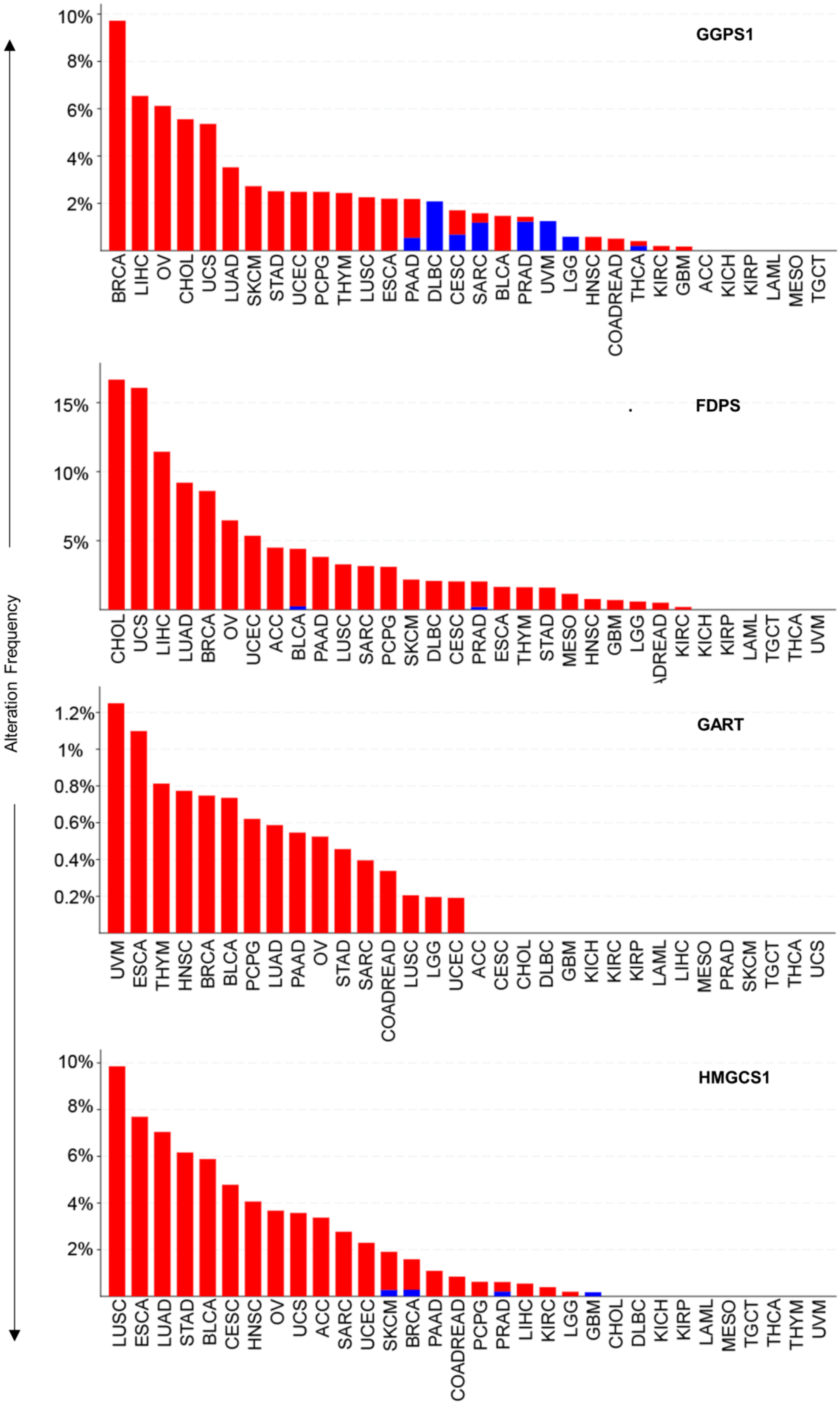

Alteration Frequency
GGPS1
10%
8%
6%
4%
2%
BRCA LIHC OV CHOL UCS LUAD SKCM STAD UCEC PCPG THYM LUSC ESCA PAAD DLBC CESC SARC BLCA PRAD UVM LGG HNSC COADREAD THCA KIRC GBM ACC KICH KIRP LAML MESO TGCT
FDPS
15%
10%
5%
CHOL UCS LIHC LUAD BRCA OV UCEC ACC BLCA PAAD LUSC SARC PCPG SKCM DLBC CESC PRAD ESCA THYM STAD MESO HNSC GBM LGG KIRC KICH KIRP LAML TGCT THCA UVM
GART
1.2%
1%
0.8%
0.6%
0.4%
0.2%
UVM ESCA THYM HNSC BRCA BLCA PCPG LUAD PAAD OV STAD SARC COADREAD LUSC LGG UCEC ACC CESC CHOL DLBC GBM KICH KIRC KIRP LAML LIHC MESO PRAD SKCM TGCT THCA UCS
HMGCS1
10%
8%
6%
4%
2%
LUSC ESCA LUAD STAD BLCA CESC HNSC OV UCS ACC SARC UCEC SKCM BRCA PAAD COADREAD PCPG PRAD LIHC KIRC LGG GBM CHOL DLBC KICH KIRP LAML MESO TGCT THCA THYM UVM

Supplementary Figure S6: CNV amplification of GGPS1, FDPS, GART, and HMGCS1 in representative human cancers. TCGA provisional Cell type used are LAML, Acute Myeloid Leukemia (n=200), ACC, Adrenocortical Carcinoma (n=92), BLCA, Bladder Urothelial Carcinoma (n=411), LGG, Brain Lower Grade Glioma (n=514), BRCA, Breast Invasive Carcinoma (n=1084), CESC, Cervical Squamous Cell Carcinoma and Endocervical Adenocarcinoma (n=297), CHOL, Cholangiocarcinoma (n=36), COADREAD, Colorectal Adenocarcinoma (n=594), ESCA, Esophageal Carcinoma (n=182), GBM, Glioblastoma Multiforme (n=592), HNSC, Head and Neck Squamous Cell Carcinoma (n=523), KICH, Kidney Chromophobe (n=65), KIRC, Kidney Renal Clear Cell Carcinoma (n=512), KIRP, Kidney Renal Papillary Cell Carcinoma (n=283), LIHC, Liver Hepatocellular Carcinoma (372), LUAD, Lung Adenocarcinoma (n=566), LUSC, Lung Squamous Cell Carcinoma (n=487), DLBC, Lymphoid Neoplasm Diffuse Large B-cell Lymphoma (n=48), MESO, Mesothelioma (n=87), OV, Ovarian Serous Cystadenocarcinoma (n=585), PAAD, Pancreatic Adenocarcinoma (n=184), PCPG, Pheochromocytoma and Paraganglioma (n=178), PRAD, Prostate Adenocarcinoma (n=494), SARC, Sarcoma (n=255), SKCM, Skin Cutaneous Melanoma (n=448), STAD, Stomach Adenocarcinoma (n=440), TGCT, Testicular Germ Cell Cancer (n=149), THYM, Thymoma (n=123), THCA, Thyroid Carcinoma (n=500), UCS, Uterine Carcinosarcoma (n=57), UCEC, Uterine Corpus Endometrial Carcinoma (n=529) and UVM, Uveal Melanoma (n=80).

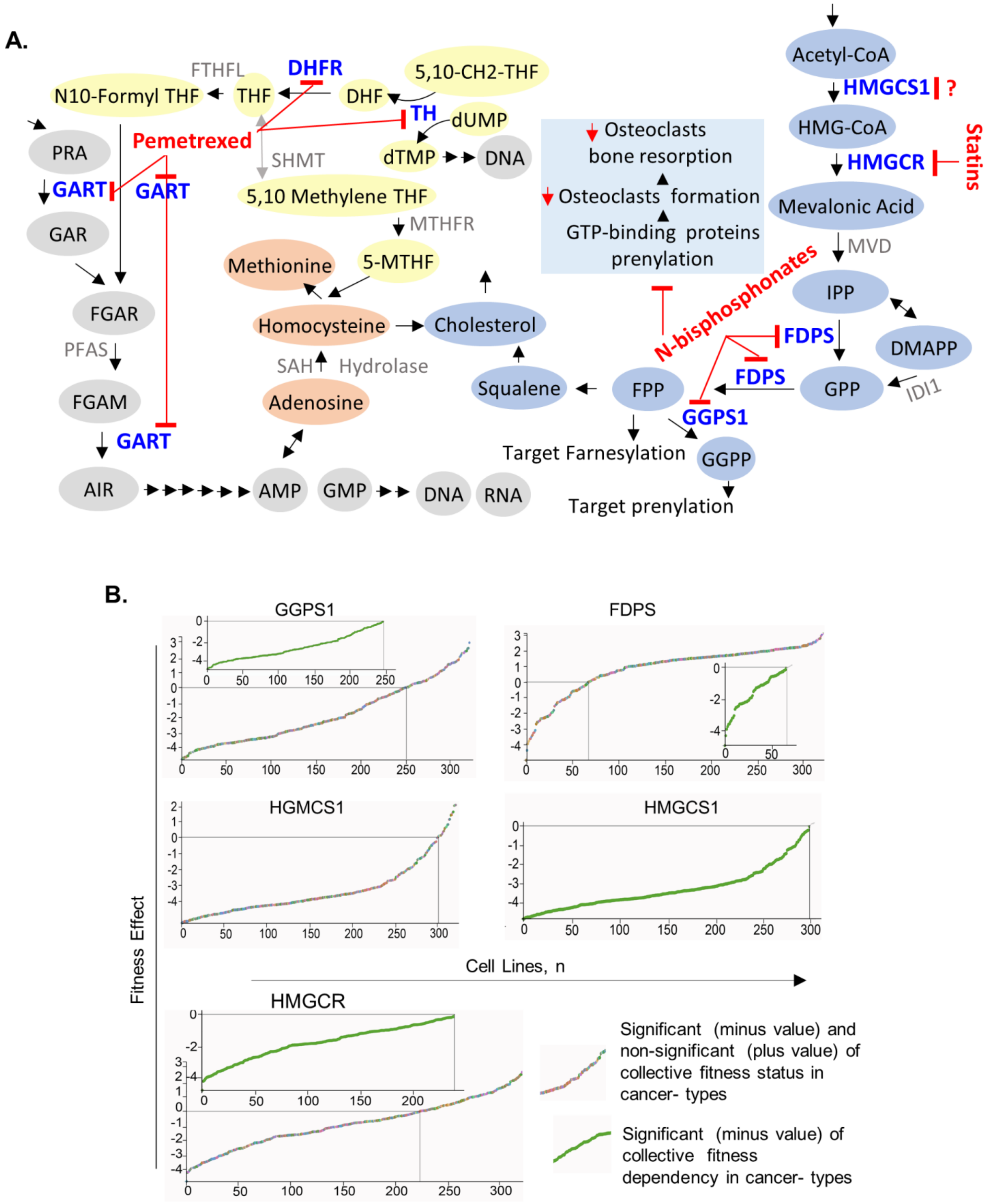
A.
N10-Formyl THF
FTHFL
THF
DHFR
DHF
5,10-CH2-THF
TH
dUMP
dTMP
DNA
Pemetrexed
PRA
GART
GART
SHMT
5,10 Methylene THF
GAR
MTHFR
5-MTHF
Methionine
FGAR
Homocysteine
Cholesterol
PFAS
SAH
Hydrolase
FGAM
Adenosine
GART
AIR
AMP
GMP
DNA
RNA
Osteoclasts bone resorption
Osteoclasts formation
GTP-binding proteins prenylation
Acetyl-CoA
HMGCS1
?
HMG-CoA
HMGCR
Statins
Mevalonic Acid
MVD
IPP
N-bisphosphonates
FDPS
DMAPP
FDPS
Squalene
FPP
GPP
IDI1
GGPS1
Target Farnesylation
GGPP
Target prenylation
B.
GGPS1
FDPS
HGMCS1
HMGCS1
HMGCR
Fitness Effect
Cell Lines, n
Significant (minus value) and non-significant (plus value) of collective fitness status in cancer- types
Significant (minus value) of collective fitness dependency in cancer- types

Supplementary Figure S7: Pathways and molecules of interest in the present study. A, Representation of molecules, enzymes and targets of N-bisphosphonates, statins, or pemetrexed. **B**, Effect of depletion of GGPS1, FDPS, HMGCS1 and HMGCR across 19 cancer types, showing significant fitness-dependency in cancer cell lines (green dots, inserts), and both significant and non-significant effects across cancer-types from Cancer Dependency Map (23) (multi-color dots, each representative a distinct cancer-type).

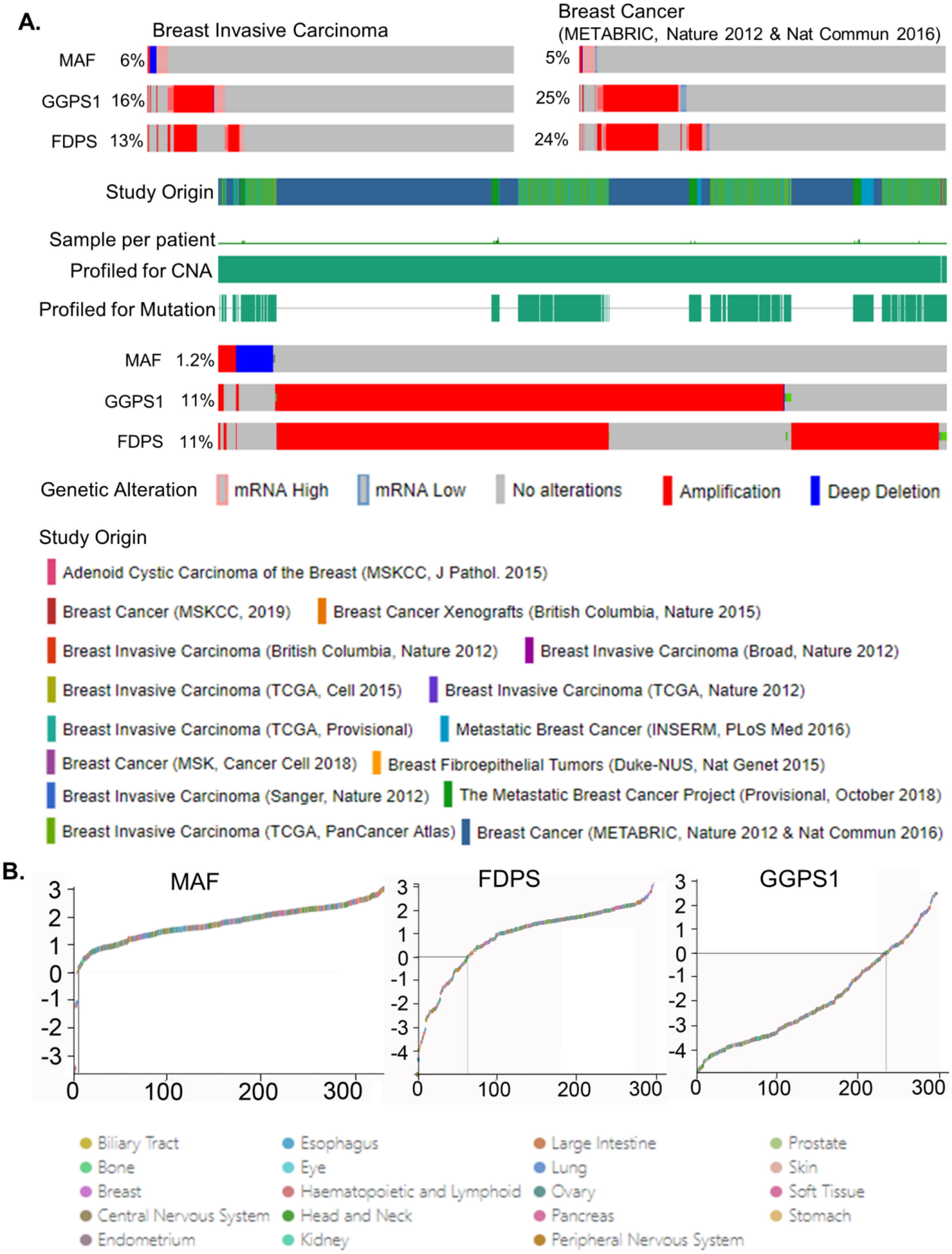
A.
Breast Invasive Carcinoma
Breast Cancer (METABRIC, Nature 2012 & Nat Commun 2016)
MAF 6%
5%
GGPS1 16%
25%
FDPS 13%
24%
Study Origin
Sample per patient
Profiled for CNA
Profiled for Mutation
MAF 1.2%
GGPS1 11%
FDPS 11%
Genetic Alteration
mRNA High
mRNA Low
No alterations
Amplification
Deep Deletion
Study Origin
Adenoid Cystic Carcinoma of the Breast (MSKCC, J Pathol. 2015)
Breast Cancer (MSKCC, 2019)
Breast Cancer Xenografts (British Columbia, Nature 2015)
Breast Invasive Carcinoma (British Columbia, Nature 2012)
Breast Invasive Carcinoma (Broad, Nature 2012)
Breast Invasive Carcinoma (TCGA, Cell 2015)
Breast Invasive Carcinoma (TCGA, Nature 2012)
Breast Invasive Carcinoma (TCGA, Provisional)
Metastatic Breast Cancer (INSERM, PLoS Med 2016)
Breast Cancer (MSK, Cancer Cell 2018)
Breast Fibroepithelial Tumors (Duke-NUS, Nat Genet 2015)
Breast Invasive Carcinoma (Sanger, Nature 2012)
The Metastatic Breast Cancer Project (Provisional, October 2018)
Breast Invasive Carcinoma (TCGA, PanCancer Atlas)
Breast Cancer (METABRIC, Nature 2012 & Nat Commun 2016)
B.
MAF
FDPS
GGPS1
3
2
1
0
-1
-2
-3
-4
0
100
200
300
Biliary Tract
Bone
Breast
Central Nervous System
Endometrium
Esophagus
Eye
Haematopoietic and Lymphoid
Head and Neck
Kidney
Large Intestine
Lung
Ovary
Pancreas
Peripheral Nervous System
Prostate
Skin
Soft Tissue
Stomach

Supplementary Figure S8: An inverse relationship between the levels of MAF versus GGPS1 or FDPS. A, Expression of MAF, GGPS1 and FDPS in breast cancer datasets. **B**, Status of fitness-dependency of MAF across cancer-types.

Dataset GSE31519: TNBC, n=67

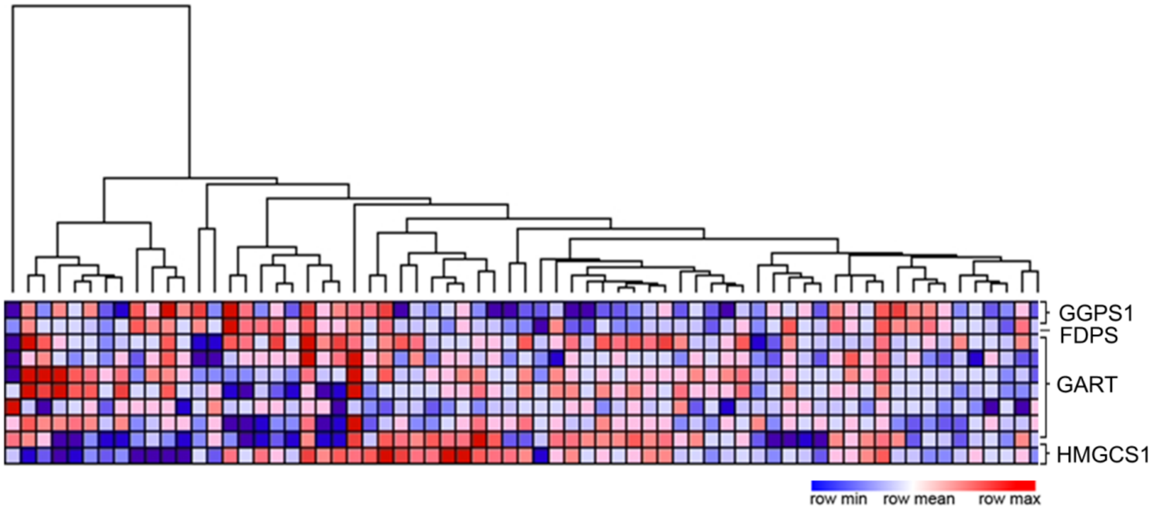

Dataset GSE58812: TNBC, n=107

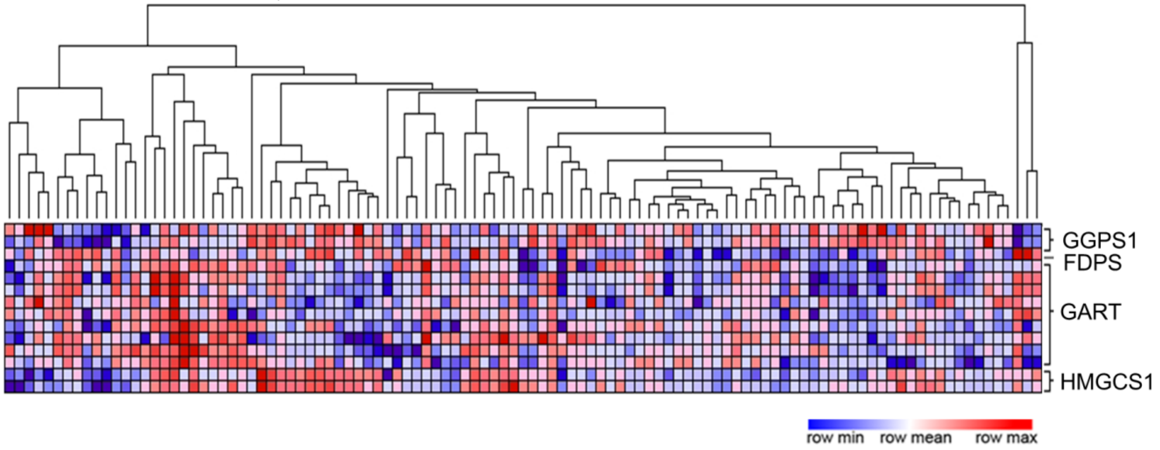

Dataset GSR83937: TNBC, n=131

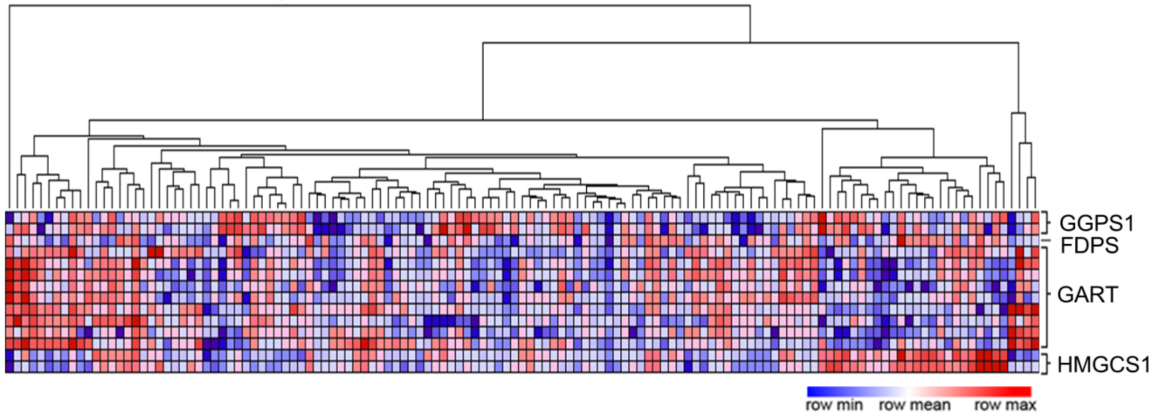

Supplementary Figure S9: Expression of GGPS1, FDPS, GART and HMGCS1 in different TNBC studies. Representation of gene expression as heatmaps in TNBC samples using GEO datasets GSE31519 (70-73), GSE58812 (74) and GSE83937 (75).

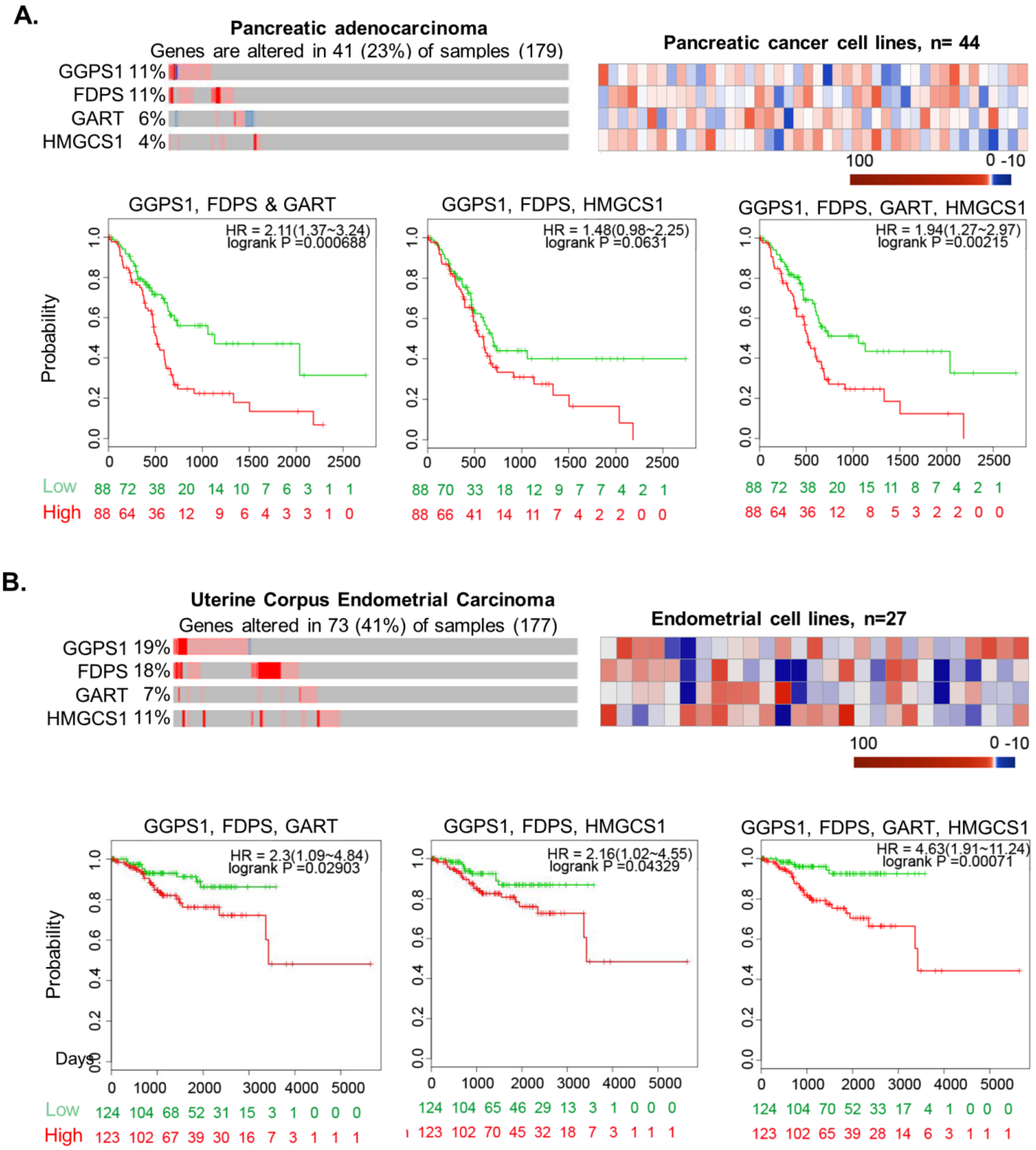
A.
Pancreatic adenocarcinoma
Genes are altered in 41 (23%) of samples (179)
GGPS1 11%
FDPS 11%
GART 6%
HMGCS1 4%
Pancreatic cancer cell lines, n= 44
100
0 -10
GGPS1, FDPS & GART
HR = 2.11(1.37~3.24)
logrank P =0.000688
GGPS1, FDPS, HMGCS1
HR = 1.48(0.98~2.25)
logrank P =0.0631
GGPS1, FDPS, GART, HMGCS1
HR = 1.94(1.27~2.97)
logrank P =0.00215
Probability
Low 88 72 38 20 14 10 7 6 3 1 1
High 88 64 36 12 9 6 4 3 3 1 0
88 70 33 18 12 9 7 7 4 2 1
88 66 41 14 11 7 4 2 2 0 0
88 72 38 20 15 11 8 7 4 2 1
88 64 36 12 8 5 3 2 2 0 0
B.
Uterine Corpus Endometrial Carcinoma
Genes altered in 73 (41%) of samples (177)
GGPS1 19%
FDPS 18%
GART 7%
HMGCS1 11%
Endometrial cell lines, n=27
100
0 -10
GGPS1, FDPS, GART
HR = 2.3(1.09~4.84)
logrank P =0.02903
GGPS1, FDPS, HMGCS1
HR = 2.16(1.02~4.55)
logrank P =0.04329
GGPS1, FDPS, GART, HMGCS1
HR = 4.63(1.91~11.24)
logrank P =0.00071
Probability
Days
Low 124 104 68 52 31 15 3 1 0 0 0
High 123 102 67 39 30 16 7 3 1 1 1
124 104 65 46 29 13 3 1 0 0 0
123 102 70 45 32 18 7 3 1 1 1
124 104 70 52 33 17 4 1 0 0 0
123 102 65 39 28 14 6 3 1 1 1

Supplementary Figure S10: Expression of GGPS1, FDPS, GART and HMGCS1 in hard-to-treat cancers. A, Amplification and expression of indicated molecules in Pancreatic CNV/mRNA [top left panel] using data from cBioPortal (29, 30) of genes in Cell lines - ASPC1, BXPC3, CAPAN1, CAPAN2, CFPAC1, DANG, HPAC, HPAFII, HS766T, HUPT3, HUPT4, KCIMOH1, KLM1, KP2, KP3, KP4, L33, MIAPACA2, PANC0203, PANC0213, PANC0327, PANC0403, PANC0504, PANC0813, PANC1005, PANC1, PATU8902, PATU8988S, PATU8988T, PK1, PK45H, PK59, PL45, PSN1, QGP1, SNU213, SNU324, SNU410, SU8686, SUIT2, SW1990, T3M4, TCCPAN2, YAPC heatmap [top right panel]. Survival plot generated using SurvExpress (32) for GGPS1/FDPS/GART/, GGPS1/FDPS/HMGCS1 and GGPS1/FDPS/GART/HMGCS1 using data from cBioPortal (29, 30). **B**, Uterine Corpus Endometrial Carcinoma CNV/mRNA [top left panel] using data from cBioPortal (29, 30). Expression of genes in Celllines-AN3CA, COLO684, EFE184, EN, ESS1, HEC108, HEC151, HEC1A, HEC1B, HEC251, HEC265, HEC50B, HEC59, HEC6, ISHIKAWAHERAKLIO02ER, JHUEM1, JHUEM2, JHUEM3, KLE, MFE280, MFE296, MFE319, RL952, SNGM, SNU1077, SNU685, TEN heatmap [top right panel]. Survival plot generated using SurvExpress (32) for GGPS1/FDPS/GART/, GGPS1/FDPS/HMGCS1 and GGPS1/FDPS/GART/HMGCS1 using data from cBioPortal (29, 30).

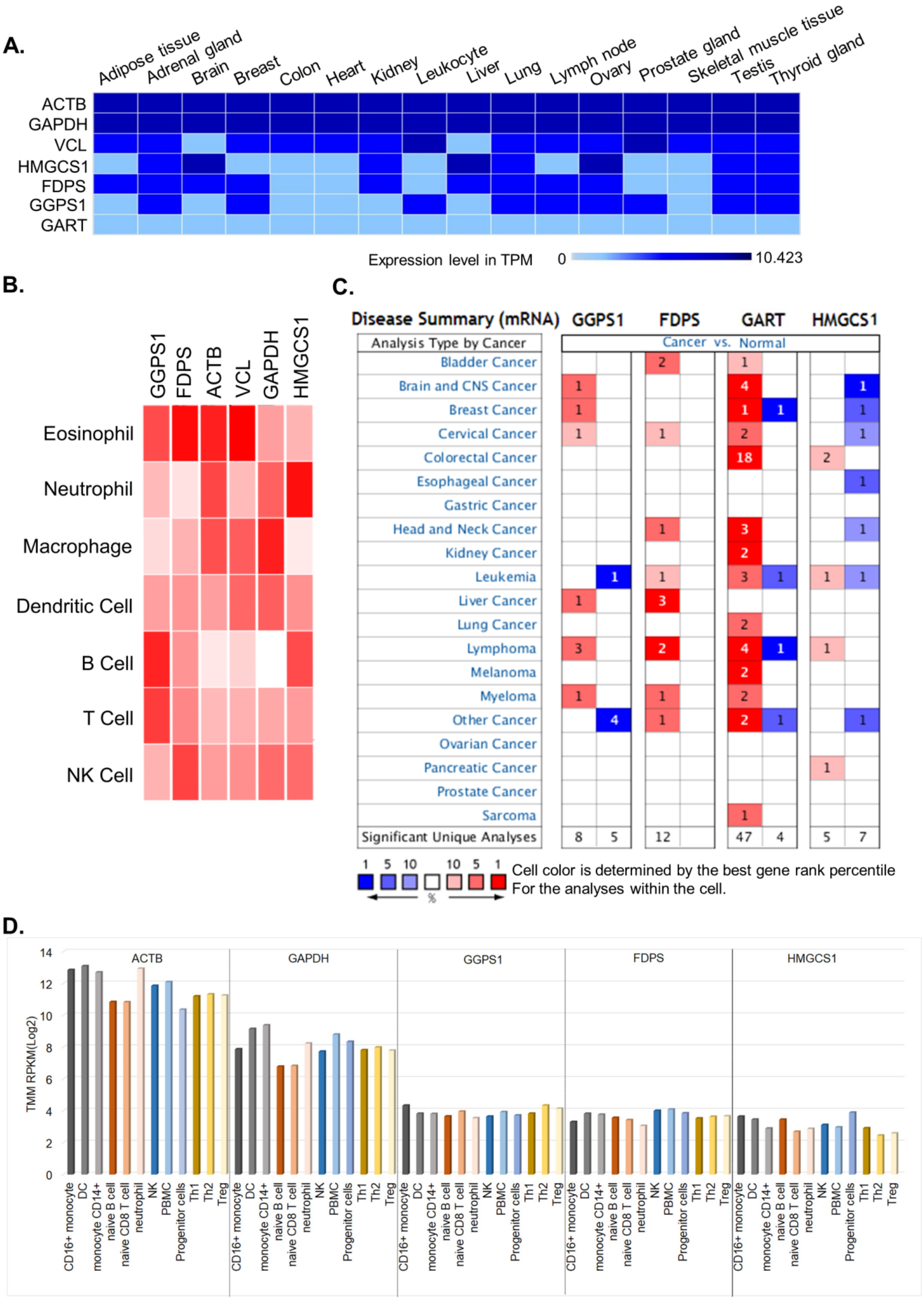
A.
Adipose tissue
Adrenal gland
Brain
Breast
Colon
Heart
Kidney
Leukocyte
Liver
Lung
Lymph node
Ovary
Prostate gland
Skeletal muscle tissue
Testis
Thyroid gland
ACTB
GAPDH
VCL
HMGCS1
FDPS
GGPS1
GART
Expression level in TPM
0
10.423
B.
GGPS1
FDPS
ACTB
VCL
GAPDH
HMGCS1
Eosinophil
Neutrophil
Macrophage
Dendritic Cell
B Cell
T Cell
NK Cell
C.
Disease Summary (mRNA)
GGPS1
FDPS
GART
HMGCS1
Analysis Type by Cancer
Cancer vs. Normal
Bladder Cancer
Brain and CNS Cancer
Breast Cancer
Cervical Cancer
Colorectal Cancer
Esophageal Cancer
Gastric Cancer
Head and Neck Cancer
Kidney Cancer
Leukemia
Liver Cancer
Lung Cancer
Lymphoma
Melanoma
Myeloma
Other Cancer
Ovarian Cancer
Pancreatic Cancer
Prostate Cancer
Sarcoma
Significant Unique Analyses
8 5 12 47 4 5 7
1 5 10 10 5 1
%
Cell color is determined by the best gene rank percentile
For the analyses within the cell.
D.
TMM RPKM(Log2)
ACTB
GAPDH
GGPS1
FDPS
HMGCS1
CD16+ monocyte
DC
monocyte CD14+
naive B cell
naive CD8 T cell
neutrophil
NK
PBMC
Progenitor cells
Th1
Th2
Treg

Supplementary Figure S11: Status of GGPS1, FDPS, GART and HMGCS1 in physiologic settings. **A,** Comparative expression analysis of GGPS1, FDPS, HMGCS1 and HMGCR against house-keeping genes, beta-action (ACTB), GAPDH and vinculin (VCL) from Illumina Body Map 2 (accession number ERP000546 in the European Nucleotide Archive). **B,** Comparative expression analysis of GGPS1, FDPS, GART and HMGCS1 in in indicated human cell-lineage using publically available RNA-Seq dataset (76).**C,** Expression of GGPS1, FDPS, GART HMGCS1 in normal cells as compared to respective cancer-types using Oncomine database (77). **D,** Status of GGPS1, FDPS and HMGCS1 mRNAs in human immune cell-types using Stemformatics (78).

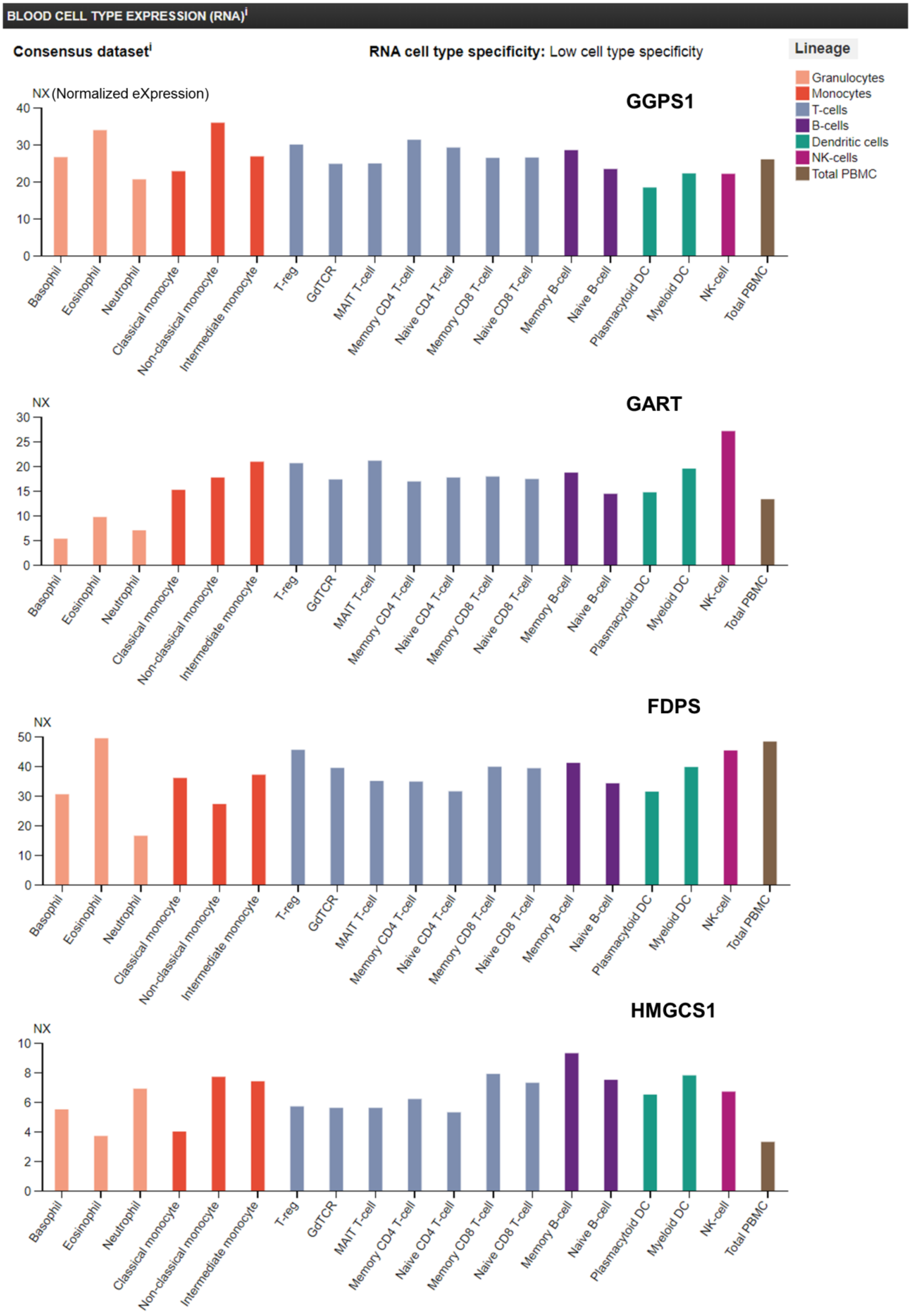
BLOOD CELL TYPE EXPRESSION (RNA)
Consensus dataset
RNA cell type specificity: Low cell type specificity
Lineage
Granulocytes
Monocytes
T-cells
B-cells
Dendritic cells
NK-cells
Total PBMC
NX (Normalized eXpression)
GGPS1
GART
FDPS
HMGCS1
NX
Basophil
Eosinophil
Neutrophil
Classical monocyte
Non-classical monocyte
Intermediate monocyte
T-reg
GdTCR
MAIT T-cell
Memory CD4 T-cell
Naive CD4 T-cell
Memory CD8 T-cell
Naive CD8 T-cell
Memory B-cell
Naive B-cell
Plasmacytoid DC
Myeloid DC
NK-cell
Total PBMC

Supplementary Figure S12: Expression of GGPS1, GART, FDPS and HMGCS1 mRNAs in blood cells using The Human Protein Atlas (Version: 19) (79).

S.NO	ACCESSION DATE (INCLUDED UPDATED)	YEAR OF FIRST APPROVAL	MONTH OF FIRST APPROVAL	YEAR OF LAST APPROVAL	MONTH OF LAST APPROVAL	DRUG	ACCESSION NUMBER	TYPE	WEIGHT (AVERAGE)
1	13.09.2019	1952	June	2019	January	Leucovorin	DB00650	Small Molecule	473.446
2	13.09.2019	1953	December	2019	April	Methotrexate	DB00563	Small Molecule	454.4393
3	13.09.2019	1954	December	2016	October	Mercaptopurine	DB01033	Small Molecule	152.177
4	13.09.2019	1956	October	2006	February	Fluoxymesterone	DB01185	Small Molecule	336.4409
5	13.09.2019	1957	December	1985	February	Chlorambucil	DB00291	Small Molecule	304.212
6	13.09.2019	1959	November	2019	May	Cyclophosphamide	DB00531	Small Molecule	261.086
7	13.09.2019	1963	December	2019	July	Dactinomycin	DB00970	Small Molecule	1255.417
8	13.09.2019	1965	November	2013	October	Vinblastine	DB00570	Small Molecule	810.9741
9	13.09.2019	1966	January	2013	March	Tioguanine	DB00352	Small Molecule	167.192
10	13.09.2019	1969	July	1985	December	Procarbazine	DB01168	Small Molecule	221.2988
11	13.09.2019	1970	December	2018	February	Floxuridine	DB00322	Small Molecule	246.1924
12	13.09.2019	1970	August	2009	June	Mitotane	DB00648	Small Molecule	320.041
13	13.09.2019	1973	December	2019	March	Bleomycin	DB00290	Small Molecule	1415.552
14	13.09.2019	1973	December	2015	September	Methyltestosterone	DB06710	Small Molecule	302.451
15	13.09.2019	1976	December	2008	March	Dacarbazine	DB00851	Small Molecule	182.187
16	13.09.2019	1976	December	2015	November	Lomustine	DB01206	Small Molecule	233.695
17	13.09.2019	1975	December	2019	September	Carmustine (Gliadel Wafer)	DB00262	Small Molecule	214.05
18	13.09.2019	1978	December	2019	April	Cisplatin	DB00515	Small Molecule	300.05
19	13.09.2019	1978	January	2016	January	Asparaginase	DB00023	Biotech (Enzyme)	31731.9 (Da)
20	13.09.2019	1981	December	1995	December	Estramustine	DB01196	Small Molecule	440.403
21	13.09.2019	1982	July	2003	March	Streptozocin	DB00428	Small Molecule	265.2206
22	13.09.2019	1983	November	2018	January	Etoposide	DB00773	Small Molecule	588.5566
23	13.09.2019	1987	August	2017	June	Ifosfamide	DB01181	Small Molecule	261.086
24	13.09.2019	1986	December	2018	February	Carboplatin	DB00958	Small Molecule	371.254
25	13.09.2019	1990	December	2006	December	Altretamine	DB00488	Small Molecule	210.2794
26	13.09.2019	1991	April	2017	December	Fludarabine	DB01073	Small Molecule	285.235
27	13.09.2019	1991	October	2007	August	Pentostatin	DB00552	Small Molecule	268.2691
28	13.09.2019	1992	December	2018	July	Paclitaxel	DB01229	Small Molecule	853.9061

29	13.09.2019	1964	December	2019	July	Melphalan	DB01042	Small Molecule	305.2
30	13.09.2019	1984	December	2013	April	Teniposide	DB00444	Small Molecule	656.654
31	13.09.2019	1993	March	2019	April	Cladribine	DB00242	Small Molecule	285.687
32	13.09.2019	1994	December	2019	August	Vinorelbine	DB00361	Small Molecule	778.947
33	13.09.2019	1994	February	2018	February	Pegasparga se	DB00059	Biotech (Enzyme)	31731.9 (Da)
34	13.09.2019	1959	December	2018	June	Thioptepa	DB04572	Small Molecule	189.218
35	13.09.2019	1995	October	2018	June	Bicalutamide	DB01128	Small Molecule	430.373
36	13.09.2019	1995	November	2019	June	Docetaxel	DB01248	Small Molecule	807.8792
37	13.09.2019	1996	May	2019	February	Gemcitabine	DB00441	Small Molecule	263.1981
38	13.09.2019	1994	December	2018	March	Goserelin (Zoladex)	DB00014	Small Molecule	1269.410 5
39	13.09.2019	1996	June	2019	Septemb er	Topotecan (Hycamtin)	DB01030	Small Molecule	421.4458
40	13.09.2019	1984	December	2016	Septemb er	Flutamide (Eulexin)	DB00499	Small Molecule	276.2118
41	13.09.2019	1996	January	2019	February	Anastrozole (Arimidex)	DB01217	Small Molecule	293.3663
42	13.09.2019	1991	November	2009	February	Pamidronic Acid (Aredia)	DB00282	Small Molecule	235.0695
43	13.09.2019	1992	December	2016	July	Nilutamide	DB00665	Small Molecule	317.2207
44	13.09.2019	1951	December	2019	March	Morphine	DB00295	Small Molecule	285.3377
45	13.09.2019	1990	December	2012	March	Ioversol	DB09134	Small Molecule	807.115
46	13.09.2019	1995	December	2009	March	Iodixanol	DB01249	Small Molecule	1550.181 9
47	13.09.2019	1990	September	2018	January	Idarubicin	DB01177	Small Molecule	497.4939
48	13.09.2019	1986	June	2014	August	Interferon alfa-2b (Intron A)	DB00105	Biotech	19271.0 (Da)
49	13.09.2019	1997	July	2019	Septemb er	Letrozole (Femara)	DB01006	Small Molecule	285.3027
50	13.09.2019	1997	February	2018	June	Imiquimod	DB00724	Small Molecule	240.3036
51	13.09.2019	1996	February	2019	January	Toremifone	DB00539	Small Molecule	405.96
52	13.09.2019	1997	May	2006	June	Samarium (1535m) lexidronam	DB05273	Small Molecule	586.021
53	13.09.2019	1998	April	2019	April	Capecitabine	DB01101	Small Molecule	359.3501
54	13.09.2019	1996	April	2018	January	Daunorubici n	DB00694	Small Molecule	527.5199
55	13.09.2019	1998	October	2019	April	Valrubicin	DB00385	Small Molecule	723.651
56	13.09.2019	1992	May	2006	May	Aldesleukin	DB00041	Biotech (Enzyme)	15314.8 (Da)
57	13.09.2019	1995	December	2006	Septemb er	Porfimer Sodium	DB00707	Small Molecule	.
58	13.09.2019	1986	December	2019	February	Tamoxifen	DB00675	Small Molecule	371.5146
59	13.09.2019	1998	July	2011	February	Thalidomide	DB01041	Small Molecule	258.2295
60	13.09.2019	1997	August	2016	July	Dolasetron	DB00757	Small Molecule	324.38
61	13.09.2019	2005	January	2019	June	Bromfenac	DB00936	Small Molecule	334.165
62	13.09.2019	1997	November	.	.	Oprelvekin	DB00038	Biotech	19047.2 (Da)
63	13.09.2019	1954	December	2017	October	Busulfan (Busulfex)	DB01008	Small Molecule	246.302
64	13.09.2019	1995	September	2019	May	Doxorubicin (Doxil)	DB00997	Small Molecule	543.5193
65	13.09.2019	1999	January	2016	January	Temozolomi de	DB00853	Small Molecule	194.1508

66	13.09.2019	1977	December	2018	September	Cytarabine	DB00987	Small Molecule	243.2166
67	13.09.2019	1999	September	2017	July	Epirubicin	DB00445	Small Molecule	543.5193
68	13.09.2019	1954	December	2016	September	Methoxsalen	DB00553	Small Molecule	216.192
69	13.09.2019	2000	October	2019	September	Alitretinoin	DB00523	Small Molecule	300.4351
70	13.09.2019	1999	December	2018	August	Bexarotene	DB00307	Small Molecule	348.4779
71	13.09.2019	1999	February	2013	November	Denileukin diftitox	DB00004	Biotech	57647.3 (Da)
72	13.09.2019	1999	October	2018	December	Exemestane	DB00990	Small Molecule	296.4034
73	13.09.2019	1995	December	2016	August	Amifostine	DB01143	Small Molecule	214.223
74	13.09.2019	2000	October	2019	September	Arsenic Trioxide	DB01169	Small Molecule	197.84
75	13.09.2019	2000	June	2017	September	Gemtuzumab ozogamicin	DB00056	Biotech	151500.0 (Da)
76	13.09.2019	2000	June	2017	September	Triptorelin	DB06825	Small Molecule	1311.473
77	13.09.2019	2006	May	2014	November	Alemtuzumab	DB00087	Biotech	145453.8 (Da)
78	13.09.2019	2001	May	2019	May	Imatinib	DB00619	Small Molecule	493.6027
79	13.09.2019	1970	July	2019	July	Fluorouracil	DB00544	Small Molecule	130.0772
80	13.09.2019	1981	August	2019	May	Mitomycin	DB00305	Small Molecule	334.3272
81	13.09.2019	2002	August	2019	June	Oxaliplatin	DB00526	Small Molecule	397.294
82	13.09.2019	2002	April	2019	September	Fulvestrant	DB00947	Small Molecule	606.78
83	13.09.2019	2002	February	2004	January	Ibritumomab tiuxetan	DB00078	Biotech	143375.5 (Da)
84	13.09.2019	1985	December	2015	July	Leuprolide	DB00007	Biotech	1209.3983 (Da)
85	13.09.2019	2002	April	2019	February	Pegfilgrastim	DB00019	Biotech	18802.8 (Da)
86	13.09.2019	1990	December	2007	June	Secretin (Human)	DB09532	Biotech	3039.44 (Da)
87	13.09.2019	2000	September	2019	January	Zoledronic Acid	DB00399	Small Molecule	272.0896
88	13.09.2019	2003	November	.		Abarelix	DB00106	Small Molecule	1416.09
89	13.09.2019	2005	May	2019	March	Bortezomib	DB00188	Small Molecule	384.237
90	13.09.2019	2003	December	2019	June	Gefitinib	DB00317	Small Molecule	446.902
91	13.09.2019	2003	June	2009	July	Tositumomab	DB00081	Biotech	143859.7 (Da)
92	13.09.2019	1957	June	2015	October	Iodide I-131	DB09293	Small Molecule	130.9067
93	13.09.2019	2003	March	2018	January	Aprepitant	DB00673	Small Molecule	534.4267
94	13.09.2019	1951	December	2015	August	Conjugated Estrogens	DB00286	Small Molecule	.
95	13.09.2019	2004	June	2019	March	Alfuzosin	DB00346	Small Molecule	389.4488
96	13.09.2019	2004	December	2019	August	Clofarabine	DB00631	Small Molecule	303.677
97	13.09.2019	2004	February	2008	October	Cetuximab	DB00002	Biotech	145781.6 (Da)
98	13.09.2019	2005	April	2019	May	Erlotinib	DB00530	Small Molecule	393.4357
99	13.09.2019	2004	July	2019	May	Azacitidine	DB00928	Small Molecule	244.2047
100	13.09.2019	2004	April	2019	June	Cinacalcet	DB01012	Small Molecule	357.412
101	13.09.2019	2006	January	2016	October	Nelarabine	DB01280	Small Molecule	297.2673

102	13.09.2019	2005	December	2007	February	Sorafenib	DB00398	Small Molecule	464.825
103	13.09.2019	2006	June	2011	September	Dasatinib	DB01254	Small Molecule	488.006
104	13.09.2019	1996	May	2014	January	Decitabine	DB01262	Small Molecule	228.2053
105	13.09.2019	2006	October	2017	January	Panitumumab	DB01269	Biotech	NA
106	13.09.2019	2006	October	2009	June	Vorinostat	DB02546	Small Molecule	264.3202
107	13.09.2019	2007	October	2017	October	Ixabepilone	DB04845	Small Molecule	506.7
108	13.09.2019	2007	March	2016	August	Lapatinib	DB01259	Small Molecule	581.058
109	13.09.2019	2007	October	2019	May	Nilotinib	DB04868	Small Molecule	529.5158
110	13.09.2019	2007	July	2019	August	Temsirolimus	DB06287	Small Molecule	1030.2871
111	13.09.2019	1998	January	2013	November	Raloxifene	DB00481	Small Molecule	473.583
112	13.09.2019	2008	March	2019	June	Bendamustine	DB06769	Small Molecule	358.263
113	13.09.2019	2009	February	2009	November	Degarelix	DB06699	Small Molecule	1632.29
114	13.09.2019	2004	February	2018	September	Pemetrexed	DB00642	Small Molecule	427.4106
115	13.09.2019	2008	January	2019	May	Fosaprepitant	DB06717	Small Molecule	614.4066
116	13.09.2019	2009	September	2019	January	Pralatrexate	DB06813	Small Molecule	477.4726
117	13.09.2019	2009	October	2016	February	Ofatumumab	DB06650	Biotech	146100.0 (Da)
118	13.09.2019	2009	November	2018	July	Romidepsin	DB06176	Small Molecule	540.69
119	13.09.2019	2001	February	2006	June	Rasburicase	DB00049	Biotech (Enzyme)	34109.5 (Da)
120	13.09.2019	1997	November	2019	May	Rituximab (Rituxan)	DB00073	Biotech	143859.7 (Da)
121	13.09.2019	1998	September	2019	July	Trastuzumab	DB00072	Biotech	145531.5 (Da)
122	13.09.2019	2010	June	2011	August	Cabazitaxel	DBO6772	Small Molecule	835.9324
123	13.09.2019	2010	November	2012	March	Eribulin	DB08871	Small Molecule	729.8966
124	13.09.2019	2010	April	.		Sipuleucel-T	DB06688	Biotech	.
125	13.09.2019	1991	February	2019	May	Ondansetron	DB00904	Small Molecule	293.363
126	13.09.2019	2001	January	2014	August	Peginterferon alfa-2b	DB00022	Biotech	31000.0 (Da)
127	13.09.2019	2006	January	2014	July	Sunitinib	DB01268	Small Molecule	398.4738
128	13.09.2019	2011	April	2019	July	Abiraterone	DB05812	Small Molecule	349.509
129	13.09.2019	2011	August	2013	February	Brentuximab vedotin	DB08870	Biotech	150500.0 (Da)
130	13.09.2019	2011	August	2012	October	Crizotinib	DB08865	Small Molecule	450.337
131	13.09.2019	2011	March	2012	March	Ipilimumab	DB06186	Biotech	148000.0 Da
132	13.09.2019	2011	February	2012	February	Vandetanib	DB05294	Small Molecule	306.365
133	13.09.2019	2011	August	2012	March	Vemurafenib	DB08881	Small Molecule	475.354
134	13.09.2019	1984	December	2013	January	Vincristine	DB00541	Small Molecule	824.972
135	13.09.2019	2012	November	.		Omacetaxine mepesuccinate	DB04865	Small Molecule	545.6213
136	13.09.2019	2009	March	2016	April	Everolimus	DB01590	Small Molecule	958.24
137	13.09.2019	2009	October	2016	July	Pazopanib	DB06589	Small Molecule	437.518

138	13.09.2019	2012	June	2013	May	Pertuzumab	DB06366	Small Molecule	489.922
139	13.09.2019	2012	January	2012	Septemb er	Axitinib	DB06626	Small Molecule	148000.0 Da
140	13.09.2019	2012	September	2017	October	Bosutinib	DB06616	Small Molecule	386.47
141	13.09.2019	2012	July	2018	May	Carfilzomib	DB08889	Small Molecule	501.514
142	13.09.2019	2012	December	2016	January	Ponatinib	DB08901	Small Molecule	719.9099
143	13.09.2019	2012	January	2013	August	Vismodegib	DB08828	Small Molecule	482.815
144	13.09.2019	2011	November	2014	May	Aflibercept	DB08885	Biotech	421.297
145	13.09.2019	2012	August	2013	June	Enzalutamid e	DB08899	Small Molecule	464.436
146	13.09.2019	1992	December	2019	March	Filgrastim	DB00099	Biotech	18800.0 (Da)
147	13.09.2019	2012	January	2013	March	Ingenol mebutate	DB05013	Small Molecule	430.541
148	13.09.2019	1984	July	2019	January	Fentanyl	DB00813	Small Molecule	336.4705
149	13.09.2019	2013	May	2017	June	Radium Ra 223 Dichloride	DB08913	Small Molecule	293.924
150	13.09.2019	2005	December	2016	Decembe r	Lenalidomid e	DB00480	Small Molecule	259.2606
151	13.09.2019	2012	September	2013	August	Regorafenib	DB08896	Small Molecule	532.5595
152	13.09.2019	2013	June	2016	April	Dabrafenib	DB08912	Small Molecule	115000.0 Da
153	13.09.2019	2013	June	2016	March	Trametinib	DB08911	Small Molecule	519.562
154	13.09.2019	2013	November	2014	Novembe r	Obinutuzum ab	DB08935	Biotech	615.3948
155	13.09.2019	2013	February	2018	October	Trastuzumab emtansine	DB05773	Biotech	146100.0 Da
156	13.09.2019	2013	July	2014	January	Afatinib	DB08916	Small Molecule	.
157	13.09.2019	2013	February	2014	February	Pomalidomid e	DB08910	Small Molecule	440.507
158	13.09.2019	1949	March	2018	Novembe r	Mechloretha mine	DB00888	Small Molecule	156.054
159	13.09.2019	2004	February	2018	August	Bevacizuma b	DB00112	Biotech	149000.0 (Da)
160	13.09.2019	2010	June	2011	July	Denosumab	DB06643	Biotech	144700.0 (Da)
161	13.09.2019	2014	July	2015	April	Idelalisib	DB09054	Small Molecule	273.2441
162	13.09.2019	2014	July	.	.	Belinostat	DB05015	Small Molecule	415.432
163	13.09.2019	2014	April	2019	March	Ceritinib	DB09063	Small Molecule	318.35
164	13.09.2019	2014	April	2015	Septemb er	Ramuciruma b	DB05578	Biotech	.
165	13.09.2019	2014	December	2016	March	Blinatumoma b	DB09052	Biotech	1096.33
166	13.09.2019	2014	October	2018	May	Olaparib	DB09074	Small Molecule	143597.3 811 Da
167	13.09.2019	2014	October	2017	Novembe r	Netupitant (+ Palonosetro n)	DB09048	Small Molecule	578.603
168	13.09.2019	2005	March	2017	Novembe r	Palonosetro n (+ Netupitant)	DB00377	Small Molecule	296.414
169	13.09.2019	1996	June	2017	October	Irinotecan	DB00762	Small Molecule	586.678
170	13.09.2019	2015	December	2016	October	Alectinib	DB11363	Small Molecule	868.45
171	13.09.2019	2015	November	2016	April	Cobimetinib	DB05239	Small Molecule	482.6166

172	13.09.2019	2015	November	2016	July	Daratumumab	DB09331	Biotech	531.318
173	13.09.2019	2015	November	2016	May	Elotuzumab	DB06317	Biotech	148100.0 (Da)
174	13.09.2019	2015	February	2015	August	Panobinostat	DB06603	Small Molecule	349.434
175	13.09.2019	2015	February	2017	May	Palbociclib	DB09073	Small Molecule	447.5328
176	13.09.2019	2015	November	.	.	Talimogene laherparepvec	DB13896	Biotech	.
177	13.09.2019	1980	April	2018	March	Trifluridine (+ Tipiracil)	DB00432	Small Molecule	296.1999
178	13.09.2019	2015	September	2018	March	Tipiracil (+ Trifluridine)	DB09343	Small Molecule	242.662
179	13.09.2019	2015	November	2016	September	Ixazomib	DB09570	Small Molecule	361.03
180	13.09.2019	2015	August	2017	September	Sonidegib	DB09143	Small Molecule	485.507
181	13.09.2019	2015	November	.	.	Necitumumab	DB09559	Biotech	.
182	13.09.2019	2015	November	2016	July	Osimertinib	DB09330	Small Molecule	499.619
183	13.09.2019	2015	September	2019	May	Dinutuximab	DB09077	Biotech	145000.0 (Da)
184	13.09.2019	2015	October	2017	October	Rolapitant	DB09291	Small Molecule	500.485
185	13.09.2019	2015	September	2016	March	Uridine triacetate	DB09144	Small Molecule	370.314
186	13.09.2019	2010	August	2015	October	Trabectedin	DB05109	Small Molecule	761.837
187	13.09.2019	2012	November	2018	October	Cabozantinib	DB08875	Small Molecule	530.446
188	13.09.2019	2016	October	2017	December	Olaratumab	DB06043	Biotech	320.396
189	13.09.2019	2015	February	2018	August	Lenvatinib	DB09078	Small Molecule	154000.0 Da
190	13.09.2019	2016	December	2017	May	Rucaparib	DB12332	Small Molecule	426.86
191	13.09.2019	1993	December	2016	August	Granisetron	DB00889	Small Molecule	323.371
192	13.09.2019	1994	August	2017	July	Dronabinol	DB00470	Small Molecule	312.417
193	13.09.2019	2016	April	2016	October	Venetoclax	DB11581	Small Molecule	314.4617
194	13.09.2019	2013	November	2018	February	Ibrutinib	DB09053	Small Molecule	485.938
195	13.09.2019	2017	September	.	.	Copanlisib	DB12483	Small Molecule	552.724
196	13.09.2019	2017	April	2018	August	Brigatinib	DB12267	Small Molecule	480.529
197	13.09.2019	2017	March	2017	December	Avelumab	DB11945	Biotech	584.1
198	13.09.2019	2017	August	2018	May	Inotuzumab ozogamicin	DB05889	Biotech	142.0 Da
199	13.09.2019	2017	October	.	.	Acalabrutinib	DB11703	Small Molecule	.
200	13.09.2019	2017	August	2019	February	Enasidenib	DB13874	Small Molecule	465.517
201	13.09.2019	2017	May	2017	November	Durvalumab	DB11714	Biotech	473.383
202	13.09.2019	2017	March	.		Ribociclib	DB11730	Small Molecule	146300.0 Da
203	13.09.2019	2017	August	2019	May	Tisagenlecleucel	DB13881	Biotech	434.548
204	13.09.2019	2017	July	.	.	Neratinib	DB11828	Small Molecule	
205	13.09.2019	2017	April	.	.	Midostaurin	DB06595	Small Molecule	557.05
206	13.09.2019	2017	September	2019	July	Abemaciclib	DB12001	Small Molecule	570.649
207	13.09.2019	1971	December	2017	August	Daunorubicin (+ Cytarabine)	DB00694	Small Molecule	506.606

208	13.09.2019	1977	December	2017	August	Cytarabine (+ Daunorubicin)	DB00987	Small Molecule	527.5199
209	13.09.2019	2017	March	2018	December	Telotristat ethyl	DB12095	Small Molecule	243.2166
210	13.09.2019	2017	October	.	.	Axicabtagene ciloleucel	DB13915	Biotech	574.99
211	13.09.2019	2017	March	.	.	Niraparib	DB11793	Small Molecule	.
212	13.09.2019	2014	December	2017	December	Nivolumab	DB09035	Biotech	54100.0 Da
213	13.09.2019	2019	August	.	.	Calaspargase pegol	DB14730	Biotech (Enzyme)	145000.0 Da
214	13.09.2019	2018	June	.	.	Encorafenib (+ Binimetinib)	DB11718	Small Molecule	.
215	13.09.2019	2018	June	.	.	Binimetinib (+ Encorafenib)	DB11967	Small Molecule	540.01
216	13.09.2019	2018	September	.	.	Duvelisib	DB11952	Small Molecule	441.233
217	13.09.2019	2018	December	.	.	Glasdegib	DB11978	Small Molecule	416.87
218	13.09.2019	2019	January	.	.	Tagraxofusp	DB14731	Biotech	374.448
219	13.09.2019	2018	February	2018	July	Apalutamide	DB11901	Small Molecule	.
220	13.09.2019	2018	September	2019	May	Cemiplimab	DB14707	Biotech	477.44
221	13.09.2019	2018	November	2019	April	Lorlatinib	DB12130	Small Molecule	146000.0 Da
222	13.09.2019	2018	October	.	.	Moxetumomab pasudotox	DB12688	Biotech	406.421
223	13.09.2019	2018	January	.	.	Lutetium Lu 177 dotatate	DB13985	Small Molecule	63500.0 Da
224	13.09.2019	2018	August	.	.	Mogamulizumab	DB12498	Biotech	1609.55
225	13.09.2019	2018	October	.	.	Talazoparib	DB11760	Small Molecule	.
226	13.09.2019	2018	July	.	.	Ivosidenib	DB14568	Small Molecule	380.359
227	13.09.2019	2018	November	2019	July	Larotrectinib	DB14723	Small Molecule	582.97
228	13.09.2019	2018	October	2019	April	Dacomitinib	DB11963	Small Molecule	428.444
229	13.09.2019	2018	November	.	.	Gilteritinib	DB12141	Small Molecule	469.939
230	13.09.2019	2014	September	2017	July	Pembrolizumab	DB09037	Biotech	558.135
231	13.09.2019	2019	April	.	.	Erdafitinib	DB12147	Small Molecule	434.4628
232	13.09.2019	2016	May	2019	March	Atezolizumab	DB11595	Biotech	446.555
233	13.09.2019	1979	December	2018	February	Hydroxyurea	DB01005	Small Molecule	76.0547
234	13.09.2019	2011	November	2015	April	Ruxolitinib	DB08877	Small Molecule	.
235	13.09.2019	2007	February	2007	November	Lanreotide	DB06791	Small Molecule	143600.0 Da

Supplementary Table S1: The list of 235 FDA approved drug targets for cancer treatment

S.NO	GENE	DRUGS ASSOCIATED WITH THE DRUG TARGETS	
1	ABL1	Vandetanib	https://www.drugbank.ca/drugs/DB05294
		Bosutinib	https://www.drugbank.ca/drugs/DB06616
		Ponatinib	https://www.drugbank.ca/drugs/DB08901
		Brigatinib	https://www.drugbank.ca/drugs/DB12267
		Dasatinib	https://www.drugbank.ca/drugs/DB01254
		Nilotinib	https://www.drugbank.ca/drugs/DB04868
2	ACPP	Sipuleucel-T	https://www.drugbank.ca/drugs/DB06688
3	ADA	Pentostatin	https://www.drugbank.ca/drugs/DB00552
		Cladribine	https://www.drugbank.ca/drugs/DB00242
4	ADRA1A	Alfuzosin	https://www.drugbank.ca/drugs/DB00346
5	ADRA1B	Alfuzosin	https://www.drugbank.ca/drugs/DB00346
6	ADRA1D	Alfuzosin	https://www.drugbank.ca/drugs/DB00346
7	AHR	Flutamide (Eulexin)	https://www.drugbank.ca/drugs/DB00499
8	AKT1	Arsenic Trioxide	https://www.drugbank.ca/drugs/DB01169
9	ALK	Crizotinib	https://www.drugbank.ca/drugs/DB08865
		Ceritinib	https://www.drugbank.ca/drugs/DB09063
		Lorlatinib	https://www.drugbank.ca/drugs/DB12130
		Gilteritinib	https://www.drugbank.ca/drugs/DB12141
		Brigatinib	https://www.drugbank.ca/drugs/DB12267
		Alectinib	https://www.drugbank.ca/drugs/DB11363
10	AR	Flutamide (Eulexin)	https://www.drugbank.ca/drugs/DB00499
		Fluoxymesterone	https://www.drugbank.ca/drugs/DB01185

		Methyltestosterone	https://www.drugbank.ca/drugs/DB06710
		Bicalutamide	https://www.drugbank.ca/drugs/DB01128
		Nilutamide	https://www.drugbank.ca/drugs/DB00665
		Apalutamide	https://www.drugbank.ca/drugs/DB11901
		Enzalutamide	https://www.drugbank.ca/drugs/DB08899
11	ARL2	Tagraxofusp	https://www.drugbank.ca/drugs/DB14731
12	ASRGL1	Asparaginase	https://www.drugbank.ca/drugs/DB00023
		Pegaspargase	https://www.drugbank.ca/drugs/DB00059
		Calaspargase pegol	https://www.drugbank.ca/drugs/DB14730
13	ATIC	Methotrexate	https://www.drugbank.ca/drugs/DB00563
		Pemetrexed	https://www.drugbank.ca/drugs/DB00642
14	ATOX1	Cisplatin	https://www.drugbank.ca/drugs/DB00515
15	AXL	Gilteritinib	https://www.drugbank.ca/drugs/DB12141
16	BCL2	Paclitaxel	https://www.drugbank.ca/drugs/DB01229
		Docetaxel	https://www.drugbank.ca/drugs/DB01248
		Eribulin	https://www.drugbank.ca/drugs/DB08871
		Venetoclax	https://www.drugbank.ca/drugs/DB11581
17	BCR	Bosutinib	https://www.drugbank.ca/drugs/DB06616
		Ponatinib	https://www.drugbank.ca/drugs/DB08901
18	BCR/ABL fusion	Imatinib	https://www.drugbank.ca/drugs/DB00619
19	BRAF	Sorafenib	https://www.drugbank.ca/drugs/DB00398
		Vemurafenib	https://www.drugbank.ca/drugs/DB08881
		Regorafenib	https://www.drugbank.ca/drugs/DB08896
		Dabrafenib	https://www.drugbank.ca/drugs/DB08912
		Encorafenib (+ Binimetinib)	https://www.drugbank.ca/drugs/DB11718
20	BTK	Ibrutinib	https://www.drugbank.ca/drugs/DB09053
		Acalabrutinib	https://www.drugbank.ca/drugs/DB11703
21	CAMK2G	Abiraterone	https://www.drugbank.ca/drugs/DB05812
22	CASR	Cinacalcet	https://www.drugbank.ca/drugs/DB01012

23	CCND1	Encorafenib (+ Binimetinib)	https://www.drugbank.ca/drugs/DB11718
		Dolasetron	https://www.drugbank.ca/drugs/DB00757
24	CD19	Blinatumomab	https://www.drugbank.ca/drugs/DB09052
		Tisagenlecleucel	https://www.drugbank.ca/drugs/DB13881
		Axicabtagene ciloleucel	https://www.drugbank.ca/drugs/DB13915
25	CD22	Inotuzumab ozogamicin	https://www.drugbank.ca/drugs/DB05889
		Moxetumomab pasudotox	https://www.drugbank.ca/drugs/DB12688
26	CD274	Atezolizumab	https://www.drugbank.ca/drugs/DB11595
		Avelumab	https://www.drugbank.ca/drugs/DB11945
		Durvalumab	https://www.drugbank.ca/drugs/DB11714
27	CD33	Gemtuzumab ozogamicin	https://www.drugbank.ca/drugs/DB00056
28	CD38	Daratumumab	https://www.drugbank.ca/drugs/DB09331
29	CD3D	Blinatumomab	https://www.drugbank.ca/drugs/DB09052
30	CD52	Alemtuzumab	https://www.drugbank.ca/drugs/DB00087
31	CD80	Durvalumab	https://www.drugbank.ca/drugs/DB11714
32	CDH5	Lenalidomide	https://www.drugbank.ca/drugs/DB00480
33	CDK2	Bosutinib	https://www.drugbank.ca/drugs/DB06616
34	CDK4	Ribociclib	https://www.drugbank.ca/drugs/DB11730
		Abemaciclib	https://www.drugbank.ca/drugs/DB12001
		Palbociclib	https://www.drugbank.ca/drugs/DB09073
35	CDK6	Ribociclib	https://www.drugbank.ca/drugs/DB11730
		Abemaciclib	https://www.drugbank.ca/drugs/DB12001
		Palbociclib	https://www.drugbank.ca/drugs/DB09073
36	CHD1	Epirubicin	https://www.drugbank.ca/drugs/DB00445
37	CMPK1	Gemcitabine	https://www.drugbank.ca/drugs/DB00441
38	CNR1	Dronabinol	https://www.drugbank.ca/drugs/DB00470
39	CNR2	Dronabinol	https://www.drugbank.ca/drugs/DB00470
40	CRBN	Thalidomide	https://www.drugbank.ca/drugs/DB01041
		Lenalidomide	https://www.drugbank.ca/drugs/DB00480

		Pomalidomide	https://www.drugbank.ca/drugs/DB08910
41	CSF1R	Erdafitinib	https://www.drugbank.ca/drugs/DB12147
		Vorinostat	https://www.drugbank.ca/drugs/DB02546
42	CSF3R	Pegfilgrastim	https://www.drugbank.ca/drugs/DB00019
		Filgrastim	https://www.drugbank.ca/drugs/DB00099
43	CTLA4	Ipilimumab	https://www.drugbank.ca/drugs/DB06186
44	CXCR4	Mogamulizumab	https://www.drugbank.ca/drugs/DB12498
45	CYP11B1	Mitotane	https://www.drugbank.ca/drugs/DB00648
46	CYP17A1	Abiraterone	https://www.drugbank.ca/drugs/DB05812
47	CYP19A1	Anastrozole (Arimidex)	https://www.drugbank.ca/drugs/DB01217
		Letrozole (Femara)	https://www.drugbank.ca/drugs/DB01006
		Exemestane	https://www.drugbank.ca/drugs/DB00990
48	DCK	Fludarabine	https://www.drugbank.ca/drugs/DB01073
49	DDR2	Vandetanib	https://www.drugbank.ca/drugs/DB05294
50	DHFR	Methotrexate	https://www.drugbank.ca/drugs/DB00563
		Pemetrexed	https://www.drugbank.ca/drugs/DB00642
51	DNA	Daunorubicin	https://www.drugbank.ca/drugs/DB00694
		Valrubicin	https://www.drugbank.ca/drugs/DB00385
		Uridine triacetate	https://www.drugbank.ca/drugs/DB09144
		Chlorambucil	https://www.drugbank.ca/drugs/DB00291
		Cyclophosphamide	https://www.drugbank.ca/drugs/DB00531
		Dactinomycin	https://www.drugbank.ca/drugs/DB00970
		Dacarbazine	https://www.drugbank.ca/drugs/DB00851
		Ifosfamide	https://www.drugbank.ca/drugs/DB01181
		Carboplatin	https://www.drugbank.ca/drugs/DB00958
		Carboplatin	https://www.drugbank.ca/drugs/DB00958
		Doxorubicin (Doxil)	https://www.drugbank.ca/drugs/DB00997
		Temozolomide	https://www.drugbank.ca/drugs/DB00853
		Altretamine	https://www.drugbank.ca/drugs/DB00488

		Fludarabine	https://www.drugbank.ca/drugs/DB01073
		Fluorouracil	https://www.drugbank.ca/drugs/DB00544
		Methoxsalen	https://www.drugbank.ca/drugs/DB00553
		Melphalan	https://www.drugbank.ca/drugs/DB01042
		Thalidomide	https://www.drugbank.ca/drugs/DB01041
		Lomustine	https://www.drugbank.ca/drugs/DB01206
		Carmustine (Gliadel Wafer)	https://www.drugbank.ca/drugs/DB00262
		Cisplatin	https://www.drugbank.ca/drugs/DB00515
		Bleomycin	https://www.drugbank.ca/drugs/DB00290
		Tioguanine	https://www.drugbank.ca/drugs/DB00352
		Procarbazine	https://www.drugbank.ca/drugs/DB01168
		Capecitabine	https://www.drugbank.ca/drugs/DB01101
		Streptozocin	https://www.drugbank.ca/drugs/DB00428
		Clofarabine	https://www.drugbank.ca/drugs/DB00631
		Cytarabine	https://www.drugbank.ca/drugs/DB00987
		Epirubicin	https://www.drugbank.ca/drugs/DB00445
		Nelarabine	https://www.drugbank.ca/drugs/DB01280
		Mitomycin	https://www.drugbank.ca/drugs/DB00305
		Oxaliplatin	https://www.drugbank.ca/drugs/DB00526
		Bendamustine	https://www.drugbank.ca/drugs/DB06769
		Gemcitabine	https://www.drugbank.ca/drugs/DB00441
		Mechlorethamine	https://www.drugbank.ca/drugs/DB00888
		Thiotepa	https://www.drugbank.ca/drugs/DB04572
		Trabectedin	https://www.drugbank.ca/drugs/DB05109
		Daunorubicin (+ Cytarabine)	https://www.drugbank.ca/drugs/DB00694
		Idarubicin	https://www.drugbank.ca/drugs/DB01177
		Cytarabine (+ Daunorubicin)	https://www.drugbank.ca/drugs/DB00987
		Mercaptopurine	https://www.drugbank.ca/drugs/DB01033
		Topotecan (Hycamtin)	https://www.drugbank.ca/drugs/DB01030

52	DNA polymerase catalytic subunit	Talimogene laherparepvec	https://www.drugbank.ca/drugs/DB13896
53	DNMT1	Azacitidine	https://www.drugbank.ca/drugs/DB00928
		Decitabine	https://www.drugbank.ca/drugs/DB01262
54	EBP	Tamoxifen	https://www.drugbank.ca/drugs/DB00675
55	EEF2	Moxetumomab pasudotox	https://www.drugbank.ca/drugs/DB12688
56	EGFR	Gefitinib	https://www.drugbank.ca/drugs/DB00317
		Cetuximab	https://www.drugbank.ca/drugs/DB00002
		Erlotinib	https://www.drugbank.ca/drugs/DB00530
		Panitumumab	https://www.drugbank.ca/drugs/DB01269
		Lapatinib	https://www.drugbank.ca/drugs/DB01259
		Vandetanib	https://www.drugbank.ca/drugs/DB05294
		Afatinib	https://www.drugbank.ca/drugs/DB08916
		Dacomitinib	https://www.drugbank.ca/drugs/DB11963
		Brigatinib	https://www.drugbank.ca/drugs/DB12267
		Neratinib	https://www.drugbank.ca/drugs/DB11828
		Necitumumab	https://www.drugbank.ca/drugs/DB09559
		Osimertinib	https://www.drugbank.ca/drugs/DB09330
57	ENPP1	Amifostine	https://www.drugbank.ca/drugs/DB01143
58	EPHA2	Dasatinib	https://www.drugbank.ca/drugs/DB01254
		Vandetanib	https://www.drugbank.ca/drugs/DB05294
59	ERBB2	Trastuzumab	https://www.drugbank.ca/drugs/DB00072
		Lapatinib	https://www.drugbank.ca/drugs/DB01259
		Pertuzumab	https://www.drugbank.ca/drugs/DB06366
		Trastuzumab emtansine	https://www.drugbank.ca/drugs/DB05773
		Afatinib	https://www.drugbank.ca/drugs/DB08916
		Brigatinib	https://www.drugbank.ca/drugs/DB12267
60	ERBB4	Afatinib	https://www.drugbank.ca/drugs/DB08916
		Brigatinib	https://www.drugbank.ca/drugs/DB12267
61	ESR1	Tamoxifen	https://www.drugbank.ca/drugs/DB00675

		Fluoxymesterone	https://www.drugbank.ca/drugs/DB01185
		Estramustine	https://www.drugbank.ca/drugs/DB01196
		Toremifene	https://www.drugbank.ca/drugs/DB00539
		Fulvestrant	https://www.drugbank.ca/drugs/DB00947
		Raloxifene	https://www.drugbank.ca/drugs/DB00481
		Conjugated Estrogens	https://www.drugbank.ca/drugs/DB00286
62	ESR2	Tamoxifen	https://www.drugbank.ca/drugs/DB00675
63	Estramustine binding protein	Estramustine	https://www.drugbank.ca/drugs/DB01196
64	FCGR1A	Porfimer Sodium	https://www.drugbank.ca/drugs/DB00707
		Bevacizumab	https://www.drugbank.ca/drugs/DB00112
65	FCGR2B	Bevacizumab	https://www.drugbank.ca/drugs/DB00112
66	FCGR3A	Bevacizumab	https://www.drugbank.ca/drugs/DB00112
67	FDPS	Pamidronic Acid	https://www.drugbank.ca/drugs/DB00282
		Zoledronic Acid	https://www.drugbank.ca/drugs/DB00399
68	FGF1	Pazopanib	https://www.drugbank.ca/drugs/DB06589
69	FGFR1	Sorafenib	https://www.drugbank.ca/drugs/DB00398
		Ponatinib	https://www.drugbank.ca/drugs/DB08901
		Regorafenib	https://www.drugbank.ca/drugs/DB08896
		Lenvatinib	https://www.drugbank.ca/drugs/DB09078
70	FGFR2	Thalidomide	https://www.drugbank.ca/drugs/DB01041
		Ponatinib	https://www.drugbank.ca/drugs/DB08901
		Regorafenib	https://www.drugbank.ca/drugs/DB08896
		Lenvatinib	https://www.drugbank.ca/drugs/DB09078
71	FGFR3	Pazopanib	https://www.drugbank.ca/drugs/DB06589
		Ponatinib	https://www.drugbank.ca/drugs/DB08901
		Lenvatinib	https://www.drugbank.ca/drugs/DB09078
72	FGFR4	Ponatinib	https://www.drugbank.ca/drugs/DB08901
		Lenvatinib	https://www.drugbank.ca/drugs/DB09078
73	FLT1	Lenvatinib	https://www.drugbank.ca/drugs/DB09078

		Axitinib	https://www.drugbank.ca/drugs/DB06626
		Regorafenib	https://www.drugbank.ca/drugs/DB08896
		Sunitinib	https://www.drugbank.ca/drugs/DB01268
		Sorafenib	https://www.drugbank.ca/drugs/DB00398
74	FLT3	Lenvatinib	https://www.drugbank.ca/drugs/DB09078
		Axitinib	https://www.drugbank.ca/drugs/DB06626
		Regorafenib	https://www.drugbank.ca/drugs/DB08896
		Vorinostat	https://www.drugbank.ca/drugs/DB02546
		Sorafenib	https://www.drugbank.ca/drugs/DB00398
75	FLT4	Sorafenib	https://www.drugbank.ca/drugs/DB00398
		Sunitinib	https://www.drugbank.ca/drugs/DB01268
		Axitinib	https://www.drugbank.ca/drugs/DB06626
		Regorafenib	https://www.drugbank.ca/drugs/DB08896
		Lenvatinib	https://www.drugbank.ca/drugs/DB09078
76	FRK	Vandetanib	https://www.drugbank.ca/drugs/DB05294
77	FYN	Dasatinib	https://www.drugbank.ca/drugs/DB01254
78	GABA-A receptor (anion channel)	Apalutamide	https://www.drugbank.ca/drugs/DB11901
79	Ganglioside GD2	Dinutuximab	https://www.drugbank.ca/drugs/DB09077
80	GART	Pemetrexed	https://www.drugbank.ca/drugs/DB00642
81	GGPS1	Zoledronic Acid	https://www.drugbank.ca/drugs/DB00399
82	GSR	Carmustine (Gliadel Wafer)	https://www.drugbank.ca/drugs/DB00262
83	HCK	Bosutinib	https://www.drugbank.ca/drugs/DB06616
84	HDAC1	Vorinostat	https://www.drugbank.ca/drugs/DB02546
		Romidepsin	https://www.drugbank.ca/drugs/DB06176
		Belinostat	https://www.drugbank.ca/drugs/DB05015
		Panobinostat	https://www.drugbank.ca/drugs/DB06603
85	HDAC2	Vorinostat	https://www.drugbank.ca/drugs/DB02546
		Romidepsin	https://www.drugbank.ca/drugs/DB06176
86	HDAC3	Vorinostat	https://www.drugbank.ca/drugs/DB02546

		Belinostat	https://www.drugbank.ca/drugs/DB05015
		Panobinostat	https://www.drugbank.ca/drugs/DB06603
87	HDAC6	Vorinostat	https://www.drugbank.ca/drugs/DB02546
		Belinostat	https://www.drugbank.ca/drugs/DB05015
		Panobinostat	https://www.drugbank.ca/drugs/DB06603
88	HPRT1	Mercaptopurine	https://www.drugbank.ca/drugs/DB01033
89	HSD11B2	Exemestane	https://www.drugbank.ca/drugs/DB00990
90	HTR3A	Ondansetron	https://www.drugbank.ca/drugs/DB00904
		Palonosetron (+ Netupitant)	https://www.drugbank.ca/drugs/DB00377
		Granisetron	https://www.drugbank.ca/drugs/DB00889
		Dolasetron	https://www.drugbank.ca/drugs/DB00757
91	HTR4	Ondansetron	https://www.drugbank.ca/drugs/DB00904
92	Hydroxylapatite	Pamidronic Acid (Aredia)	https://www.drugbank.ca/drugs/DB00282
		Zoledronic Acid	https://www.drugbank.ca/drugs/DB00399
93	Hydroxypatite	Radium Ra 223 Dichloride	https://www.drugbank.ca/drugs/DB08913
94	IDH1	Ivosidenib	https://www.drugbank.ca/drugs/DB14568
95	IDH2	Enasidenib	https://www.drugbank.ca/drugs/DB13874
96	IFNAR1	Interferon alfa-2b (Intron A)	https://www.drugbank.ca/drugs/DB00105
		Peginterferon alfa-2b	https://www.drugbank.ca/drugs/DB00022
97	IFNAR2	Interferon alfa-2b (Intron A)	https://www.drugbank.ca/drugs/DB00105
		Peginterferon alfa-2b	https://www.drugbank.ca/drugs/DB00022
98	IGF1R	Brigatinib	https://www.drugbank.ca/drugs/DB12267
99	IKBKB	Arsenic Trioxide	https://www.drugbank.ca/drugs/DB01169
100	IL11RA	Oprelvekin	https://www.drugbank.ca/drugs/DB00038
101	IL2RA	Aldesleukin	https://www.drugbank.ca/drugs/DB00041
		Denileukin diftitox	https://www.drugbank.ca/drugs/DB00004
102	IL2RB	Denileukin diftitox	https://www.drugbank.ca/drugs/DB00004
		Aldesleukin	https://www.drugbank.ca/drugs/DB00041
103	IL2RG	Aldesleukin	https://www.drugbank.ca/drugs/DB00041

104	IL3RA	Tagraxofusp	https://www.drugbank.ca/drugs/DB14731
105	IMPDH	Mercaptopurine	https://www.drugbank.ca/drugs/DB01033
106	ITK	Pemetrexed	https://www.drugbank.ca/drugs/DB00642
107	JAK1	Ruxolitinib	https://www.drugbank.ca/drugs/DB08877
108	JAK2	Ruxolitinib	https://www.drugbank.ca/drugs/DB08877
109	JUN	Arsenic Trioxide	https://www.drugbank.ca/drugs/DB01169
110	KCNH2	Tamoxifen	https://www.drugbank.ca/drugs/DB00675
111	KDR	Ramucirumab	https://www.drugbank.ca/drugs/DB05578
		Cabozantinib	https://www.drugbank.ca/drugs/DB08875
		Lenvatinib	https://www.drugbank.ca/drugs/DB09078
		Axitinib	https://www.drugbank.ca/drugs/DB06626
		Regorafenib	https://www.drugbank.ca/drugs/DB08896
		Ipilimumab	https://www.drugbank.ca/drugs/DB06186
		Vorinostat	https://www.drugbank.ca/drugs/DB02546
		Sorafenib	https://www.drugbank.ca/drugs/DB00398
		Midostaurin	https://www.drugbank.ca/drugs/DB06595
112	KIT	Sunitinib	https://www.drugbank.ca/drugs/DB01268
		Pazopanib	https://www.drugbank.ca/drugs/DB06589
		Ponatinib	https://www.drugbank.ca/drugs/DB08901
		Regorafenib	https://www.drugbank.ca/drugs/DB08896
		Midostaurin	https://www.drugbank.ca/drugs/DB06595
		Imatinib	https://www.drugbank.ca/drugs/DB00619
		Sorafenib	https://www.drugbank.ca/drugs/DB00398
		Dasatinib	https://www.drugbank.ca/drugs/DB01254
		Nilotinib	https://www.drugbank.ca/drugs/DB04868
113	LCK	Ponatinib	https://www.drugbank.ca/drugs/DB08901
		Dasatinib	https://www.drugbank.ca/drugs/DB01254
114	LHCGR	Goserelin (Zoladex)	https://www.drugbank.ca/drugs/DB00014
115	LIG1	Bleomycin	https://www.drugbank.ca/drugs/DB00290

116	LIG3	Bleomycin	https://www.drugbank.ca/drugs/DB00290
117	LIMK1	Omacetaxine mepesuccinate	https://www.drugbank.ca/drugs/DB04865
118	LY96	Morphine	https://www.drugbank.ca/drugs/DB00295
119	LYN	Ponatinib	https://www.drugbank.ca/drugs/DB08901
120	MAOA	Procarbazine	https://www.drugbank.ca/drugs/DB01168
121	MAP2K1	Trametinib	https://www.drugbank.ca/drugs/DB08911
		Cobimetinib	https://www.drugbank.ca/drugs/DB05239
122	MAP2K2	Trametinib	https://www.drugbank.ca/drugs/DB08911
		Binimetinib (+ Encorafenib)	https://www.drugbank.ca/drugs/DB11967
		Cobimetinib	https://www.drugbank.ca/drugs/DB05239
123	MAPK1	Arsenic Trioxide	https://www.drugbank.ca/drugs/DB01169
124	MAPK11	Vandetanib	https://www.drugbank.ca/drugs/DB05294
125	MAPK3	Dolasetron	https://www.drugbank.ca/drugs/DB00757
126	MAPKAPK2	Abiraterone	https://www.drugbank.ca/drugs/DB05812
127	MET	Crizotinib	https://www.drugbank.ca/drugs/DB08865
		Cabozantinib	https://www.drugbank.ca/drugs/DB08875
		Brigatinib	https://www.drugbank.ca/drugs/DB12267
128	Mi/protein 1 acrotubule-associated protein 2	Estramustine	https://www.drugbank.ca/drugs/DB01196
129	mRNA of Bcl-2	Cytarabine	https://www.drugbank.ca/drugs/DB00987
130	MS4A1	Ibritumomab tiuxetan	https://www.drugbank.ca/drugs/DB00078
		Tositumomab	https://www.drugbank.ca/drugs/DB00081
		Ofatumumab	https://www.drugbank.ca/drugs/DB06650
		Rituximab (Rituxan)	https://www.drugbank.ca/drugs/DB00073
		Tisagenlecleucel	https://www.drugbank.ca/drugs/DB13881
		Obinutuzumab	https://www.drugbank.ca/drugs/DB08935
131	MTOR	Temsirolimus	https://www.drugbank.ca/drugs/DB06287
		Everolimus	https://www.drugbank.ca/drugs/DB01590
132	NEK11	Dabrafenib	https://www.drugbank.ca/drugs/DB08912

133	New Bone Formation	Samarium (1535m) lexidronam	https://www.drugbank.ca/drugs/DB05273
134	NFKB1	Thalidomide	https://www.drugbank.ca/drugs/DB01041
135	NK1R	Rolapitant	https://www.drugbank.ca/drugs/DB09291
136	NR1I2	Paclitaxel	https://www.drugbank.ca/drugs/DB01229
		Erlotinib	https://www.drugbank.ca/drugs/DB00530
		Docetaxel	https://www.drugbank.ca/drugs/DB01248
137	NR3C1	Exemestane	https://www.drugbank.ca/drugs/DB00990
138	NTRK1	Vandetanib	https://www.drugbank.ca/drugs/DB05294
		Larotrectinib	https://www.drugbank.ca/drugs/DB14723
139	NTRK2	Larotrectinib	https://www.drugbank.ca/drugs/DB14723
140	NTRK3	Larotrectinib	https://www.drugbank.ca/drugs/DB14723
141	Nuclear factor kappa-light-chain-enhancer of activated B cells	Fluorouracil	https://www.drugbank.ca/drugs/DB00544
142	OPRD1	Fentanyl	https://www.drugbank.ca/drugs/DB00813
		Morphine	https://www.drugbank.ca/drugs/DB00295
143	OPRK1	Morphine	https://www.drugbank.ca/drugs/DB00295
		Fentanyl	https://www.drugbank.ca/drugs/DB00813
144	OPRM1	Fentanyl	https://www.drugbank.ca/drugs/DB00813
		Morphine	https://www.drugbank.ca/drugs/DB00295
145	ORM	Fluorouracil	https://www.drugbank.ca/drugs/DB00544
146	PARP1	Rucaparib	https://www.drugbank.ca/drugs/DB12332
		Talazoparib	https://www.drugbank.ca/drugs/DB11760
		Olaparib	https://www.drugbank.ca/drugs/DB09074
		Niraparib	https://www.drugbank.ca/drugs/DB11793
147	PARP2	Rucaparib	https://www.drugbank.ca/drugs/DB12332
		Niraparib	https://www.drugbank.ca/drugs/DB11793
		Talazoparib	https://www.drugbank.ca/drugs/DB11760
		Olaparib	https://www.drugbank.ca/drugs/DB09074

148	PARP3	Rucaparib	https://www.drugbank.ca/drugs/DB12332
		Olaparib	https://www.drugbank.ca/drugs/DB09074
		Talazoparib	https://www.drugbank.ca/drugs/DB11760
		Niraparib	https://www.drugbank.ca/drugs/DB11793
149	PDCD1	Pembrolizumab	https://www.drugbank.ca/drugs/DB09037
		Nivolumab	https://www.drugbank.ca/drugs/DB09035
		Cemiplimab	https://www.drugbank.ca/drugs/DB14707
150	PDGFRA	Sunitinib	https://www.drugbank.ca/drugs/DB01268
		Pazopanib	https://www.drugbank.ca/drugs/DB06589
		Ponatinib	https://www.drugbank.ca/drugs/DB08901
		Regorafenib	https://www.drugbank.ca/drugs/DB08896
		Midostaurin	https://www.drugbank.ca/drugs/DB06595
151	PDGFRB	Sorafenib	https://www.drugbank.ca/drugs/DB00398
		Dasatinib	https://www.drugbank.ca/drugs/DB01254
		Sunitinib	https://www.drugbank.ca/drugs/DB01268
		Pazopanib	https://www.drugbank.ca/drugs/DB06589
		Regorafenib	https://www.drugbank.ca/drugs/DB08896
		Midostaurin	https://www.drugbank.ca/drugs/DB06595
152	PGD	Dacarbazine	https://www.drugbank.ca/drugs/DB00851
153	PGF	Aflibercept	https://www.drugbank.ca/drugs/DB08885
154	PIK3CA	Copanlisib	https://www.drugbank.ca/drugs/DB12483
155	PIK3CB	Copanlisib	https://www.drugbank.ca/drugs/DB12483
156	PIK3CD	Idelalisib	https://www.drugbank.ca/drugs/DB09054
		Duvelisib	https://www.drugbank.ca/drugs/DB11952
157	PIK3CG	Duvelisib	https://www.drugbank.ca/drugs/DB11952
158	PKC	Daunorubicin	https://www.drugbank.ca/drugs/DB00694
159	PNP	Cladribine	https://www.drugbank.ca/drugs/DB00242
160	POLA1	Fludarabine	https://www.drugbank.ca/drugs/DB01073
		Cladribine	https://www.drugbank.ca/drugs/DB00242

		Clofarabine	https://www.drugbank.ca/drugs/DB00631
161	POLB	Cytarabine	https://www.drugbank.ca/drugs/DB00987
162	POLE	Cladribine	https://www.drugbank.ca/drugs/DB00242
163	POLE2	Cladribine	https://www.drugbank.ca/drugs/DB00242
164	POLE3	Cladribine	https://www.drugbank.ca/drugs/DB00242
165	POLE4	Cladribine	https://www.drugbank.ca/drugs/DB00242
166	PRKCA	Tamoxifen	https://www.drugbank.ca/drugs/DB00675
		Midostaurin	https://www.drugbank.ca/drugs/DB06595
167	PRKCD	Ingenol mebutate	https://www.drugbank.ca/drugs/DB05013
168	PRLR	Fluoxymesterone	https://www.drugbank.ca/drugs/DB01185
169	PSMB1	Ixazomib	https://www.drugbank.ca/drugs/DB09570
		Bortezomib	https://www.drugbank.ca/drugs/DB00188
170	PSMB10	Carfilzomib	https://www.drugbank.ca/drugs/DB08889
171	PSMB2	Ixazomib	https://www.drugbank.ca/drugs/DB09570
		Carfilzomib	https://www.drugbank.ca/drugs/DB08889
172	PSMB5	Ixazomib	https://www.drugbank.ca/drugs/DB09570
		Bortezomib	https://www.drugbank.ca/drugs/DB00188
173	PSMB8	Carfilzomib	https://www.drugbank.ca/drugs/DB08889
174	PSMB9	Carfilzomib	https://www.drugbank.ca/drugs/DB08889
175	PTGS1	Bromfenac	https://www.drugbank.ca/drugs/DB00963
176	PTGS2	Nelarabine	https://www.drugbank.ca/drugs/DB01280
		Bromfenac	https://www.drugbank.ca/drugs/DB00963
		Fluorouracil	https://www.drugbank.ca/drugs/DB00544
		Pomalidomide	https://www.drugbank.ca/drugs/DB08910
177	PTK6	Vandetanib	https://www.drugbank.ca/drugs/DB05294
178	RAF1	Sorafenib	https://www.drugbank.ca/drugs/DB00398
		Regorafenib	https://www.drugbank.ca/drugs/DB08896
		Dabrafenib	https://www.drugbank.ca/drugs/DB08912
179	RARA	Alitretinoin	https://www.drugbank.ca/drugs/DB00523

180	RARB	Alitretinoin	https://www.drugbank.ca/drugs/DB00523
181	RARG	Alitretinoin	https://www.drugbank.ca/drugs/DB00523
182	RET	Imatinib	https://www.drugbank.ca/drugs/DB00619
		Erdafitinib	https://www.drugbank.ca/drugs/DB12147
		Vandetanib	https://www.drugbank.ca/drugs/DB05294
		Ipilimumab	https://www.drugbank.ca/drugs/DB06186
183	Ribonucleoside-diphosphate reductase subunit	Cladribine	https://www.drugbank.ca/drugs/DB00242
184	RNA	Dactinomycin	https://www.drugbank.ca/drugs/DB00970
		Capecitabine	https://www.drugbank.ca/drugs/DB01101
		Fluorouracil	https://www.drugbank.ca/drugs/DB00544
185	RPL3	Omacetaxine mepesuccinate	https://www.drugbank.ca/drugs/DB04865
186	RRM1	Fludarabine	https://www.drugbank.ca/drugs/DB01073
		Gemcitabine	https://www.drugbank.ca/drugs/DB00441
		Clofarabine	https://www.drugbank.ca/drugs/DB00631
		Hydroxyurea	https://www.drugbank.ca/drugs/DB01005
187	RXRA	Bexarotene	https://www.drugbank.ca/drugs/DB00307
188	RXRB	Bexarotene	https://www.drugbank.ca/drugs/DB00307
189	RXRG	Bexarotene	https://www.drugbank.ca/drugs/DB00307
190	SCTR	Secretin (Human)	https://www.drugbank.ca/drugs/DB09532
191	SH2B3	Pemetrexed	https://www.drugbank.ca/drugs/DB00642
192	SIK1	Dabrafenib	https://www.drugbank.ca/drugs/DB08912
193	SLAMF7	Elotuzumab	https://www.drugbank.ca/drugs/DB06317
194	SLC2A2	Streptozocin	https://www.drugbank.ca/drugs/DB00428
195	SMO	Vismodegib	https://www.drugbank.ca/drugs/DB08828
		Sonidegib	https://www.drugbank.ca/drugs/DB09143
		Glasdegib	https://www.drugbank.ca/drugs/DB11978
196	SRC	Dasatinib	https://www.drugbank.ca/drugs/DB01254
		Bosutinib	https://www.drugbank.ca/drugs/DB06616

		Ponatinib	https://www.drugbank.ca/drugs/DB08901
197	SSTR1	Lutetium Lu 177 dotatate	https://www.drugbank.ca/drugs/DB13985
198	SSTR2	Lutetium Lu 177 dotatate	https://www.drugbank.ca/drugs/DB13985
		Lanreotide	https://www.drugbank.ca/drugs/DB06791
199	SSTR3	Lutetium Lu 177 dotatate	https://www.drugbank.ca/drugs/DB13985
200	SSTR4	Lutetium Lu 177 dotatate	https://www.drugbank.ca/drugs/DB13985
201	SSTR5	Lutetium Lu 177 dotatate	https://www.drugbank.ca/drugs/DB13985
		Lanreotide	https://www.drugbank.ca/drugs/DB06791
202	STMN4	Lomustine	https://www.drugbank.ca/drugs/DB01206
203	Substance-P receptor	Aprepitant	https://www.drugbank.ca/drugs/DB00673
204	TACR1	Fosaprepitant	https://www.drugbank.ca/drugs/DB06717
205	TEK	Sipuleucel-T	https://www.drugbank.ca/drugs/DB06688
		Ipilimumab	https://www.drugbank.ca/drugs/DB06186
		Vandetanib	https://www.drugbank.ca/drugs/DB05294
206	Thyroidal Tissue	Iodide I-131	https://www.drugbank.ca/drugs/DB09293
207	TLR7	Imiquimod	https://www.drugbank.ca/drugs/DB00724
208	TLR8	Imiquimod	https://www.drugbank.ca/drugs/DB00724
209	TNFRSF8	Brentuximab vedotin	https://www.drugbank.ca/drugs/DB08870
210	TNFSF11	Lenalidomide	https://www.drugbank.ca/drugs/DB00480
		Denosumab	https://www.drugbank.ca/drugs/DB06643
211	TOP1	Topotecan (Hycamtin)	https://www.drugbank.ca/drugs/DB01030
		Irinotecan	https://www.drugbank.ca/drugs/DB00762
212	TOP1MT	Irinotecan	https://www.drugbank.ca/drugs/DB00762
		Topotecan (Hycamtin)	https://www.drugbank.ca/drugs/DB01030
213	TOP2A	Doxorubicin	https://www.drugbank.ca/drugs/DB00997
		Etoposide	https://www.drugbank.ca/drugs/DB00773
		Teniposide	https://www.drugbank.ca/drugs/DB00444
		Idarubicin	https://www.drugbank.ca/drugs/DB01177
		Daunorubicin	https://www.drugbank.ca/drugs/DB00694

		Valrubicin	https://www.drugbank.ca/drugs/DB00385
		Epirubicin	https://www.drugbank.ca/drugs/DB00445
214	TOP2B	Etoposide	https://www.drugbank.ca/drugs/DB00773
		Daunorubicin	https://www.drugbank.ca/drugs/DB00694
215	TPH1	Telotristat ethyl	https://www.drugbank.ca/drugs/DB12095
216	TPH2	Telotristat ethyl	https://www.drugbank.ca/drugs/DB12095
217	TUBA1A	Vinblastine	https://www.drugbank.ca/drugs/DB00570
218	TUBA4A	Cabazitaxel	https://www.drugbank.ca/drugs/DB06772
		Vincristine	https://www.drugbank.ca/drugs/DB00541
219	TUBB	Vincristine	https://www.drugbank.ca/drugs/DB00541
		Vinorelbine	https://www.drugbank.ca/drugs/DB00361
220	TUBB1	Cabazitaxel	https://www.drugbank.ca/drugs/DB06772
		Paclitaxel	https://www.drugbank.ca/drugs/DB01229
		Eribulin	https://www.drugbank.ca/drugs/DB08871
221	TUBB3	Ixabepilone	https://www.drugbank.ca/drugs/DB04845
222	TUBD1	Vinblastine	https://www.drugbank.ca/drugs/DB00570
223	TUBE1	Vinblastine	https://www.drugbank.ca/drugs/DB00570
224	TUBG1	Vinblastine	https://www.drugbank.ca/drugs/DB00570
225	Tubulin	Teniposide	https://www.drugbank.ca/drugs/DB00444
		Docetaxel	https://www.drugbank.ca/drugs/DB01248
226	TXNRD1	Arsenic Trioxide	https://www.drugbank.ca/drugs/DB01169
227	TYMP	Tipiracil (+ Trifluridine)	https://www.drugbank.ca/drugs/DB09343
228	TYMS	Leucovorin	https://www.drugbank.ca/drugs/DB00650
		Methotrexate	https://www.drugbank.ca/drugs/DB00563
		Floxuridine	https://www.drugbank.ca/drugs/DB00322
		Fluorouracil	https://www.drugbank.ca/drugs/DB00544
		Gemcitabine	https://www.drugbank.ca/drugs/DB00441
		Irinotecan	https://www.drugbank.ca/drugs/DB00762
		Capecitabine	https://www.drugbank.ca/drugs/DB01101

		Pralatrexate	https://www.drugbank.ca/drugs/DB06813
		Trifluridine (+ Tipiracil)	https://www.drugbank.ca/drugs/DB00432
		Pemetrexed	https://www.drugbank.ca/drugs/DB00642
229	Uric Acid	Rasburicase	https://www.drugbank.ca/drugs/DB00049
230	VEGFA	Vandetanib	https://www.drugbank.ca/drugs/DB05294
		Aflibercept	https://www.drugbank.ca/drugs/DB08885
		Bevacizumab	https://www.drugbank.ca/drugs/DB00112
231	VEGFB	Aflibercept	https://www.drugbank.ca/drugs/DB08885
		Bevacizumab	https://www.drugbank.ca/drugs/DB00112
232	YES1	Dasatinib	https://www.drugbank.ca/drugs/DB01254

Supplementary Table S2: Drug targets and associated drug from Drugbank

S.NO	GENE	ENSEMBL GENE ID	ENTREZ ID	SANGER DEMAP PROJECT SCORE LINK
1	ADA	ENSG00000196839	100	https://score.depmap.sanger.ac.uk/gene/SIDG00356
2	AHR	ENSG00000106546	196	https://score.depmap.sanger.ac.uk/gene/SIDG00669
3	AKT1	ENSG00000142208	207	https://score.depmap.sanger.ac.uk/gene/SIDG00784
4	ALK	ENSG00000171094	238	https://score.depmap.sanger.ac.uk/gene/SIDG00858
5	AR	ENSG00000169083	367	https://score.depmap.sanger.ac.uk/gene/SIDG01312
6	ARL2	ENSG00000213465	402	https://score.depmap.sanger.ac.uk/gene/SIDG01462
7	ATIC	ENSG00000138363	471	https://score.depmap.sanger.ac.uk/gene/SIDG01760
8	ATOX1	ENSG00000177556	475	https://score.depmap.sanger.ac.uk/gene/SIDG01770
9	BCL2	ENSG00000171791	596	https://score.depmap.sanger.ac.uk/gene/SIDG02177
10	BRAF	ENSG00000157764	673	https://score.depmap.sanger.ac.uk/gene/SIDG02491
11	BTK	ENSG00000010671	695	https://score.depmap.sanger.ac.uk/gene/SIDG02622
12	CCND1	ENSG00000110092	595	https://score.depmap.sanger.ac.uk/gene/SIDG03838
13	CD19	ENSG00000177455	930	https://score.depmap.sanger.ac.uk/gene/SIDG03947
14	CD3D	ENSG00000167286	915	https://score.depmap.sanger.ac.uk/gene/SIDG03932
15	CD52	ENSG00000169442	1043	https://score.depmap.sanger.ac.uk/gene/SIDG03970
16	CDK2	ENSG00000123374	1017	https://score.depmap.sanger.ac.uk/gene/SIDG04140
17	CDK4	ENSG00000135446	1019	https://score.depmap.sanger.ac.uk/gene/SIDG04147
18	CDK6	ENSG00000105810	1021	https://score.depmap.sanger.ac.uk/gene/SIDG04156
19	CHD1	ENSG00000153922	1105	https://score.depmap.sanger.ac.uk/gene/SIDG04542
20	CMPK1	ENSG00000162368	51727	https://score.depmap.sanger.ac.uk/gene/SIDG04945
21	CRBN	ENSG00000113851	51185	https://score.depmap.sanger.ac.uk/gene/SIDG05383

22	CYP17A1	ENSG00000148795	1586	https://score.depmap.sanger.ac.uk/gene/SIDG05976
23	DHFR	ENSG00000178700	1719	https://score.depmap.sanger.ac.uk/gene/SIDG06513
24	DNMT1	ENSG00000130816	1786	https://score.depmap.sanger.ac.uk/gene/SIDG06847
25	EBP	ENSG00000147155	10682	https://score.depmap.sanger.ac.uk/gene/SIDG07262
26	EGFR	ENSG00000146648	1956	https://score.depmap.sanger.ac.uk/gene/SIDG07457
27	ERBB2	ENSG00000141736	2064	https://score.depmap.sanger.ac.uk/gene/SIDG07927
28	ERBB4	ENSG00000178568	2066	https://score.depmap.sanger.ac.uk/gene/SIDG07929
29	ESR1	ENSG00000091831	2099	https://score.depmap.sanger.ac.uk/gene/SIDG08129
30	ESR2	ENSG00000140009	2100	https://score.depmap.sanger.ac.uk/gene/SIDG08130
31	FCGR1A	ENSG00000150337	2209	https://score.depmap.sanger.ac.uk/gene/SIDG08944
32	FCGR2B	ENSG00000072694	2213	https://score.depmap.sanger.ac.uk/gene/SIDG08948
33	FCGR3A	ENSG00000203747	2214	https://score.depmap.sanger.ac.uk/gene/SIDG08950
34	FDPS	ENSG00000160752	2224	https://score.depmap.sanger.ac.uk/gene/SIDG08977
35	FGF1	ENSG00000113578	2246	https://score.depmap.sanger.ac.uk/gene/SIDG09045
36	FGFR1	ENSG00000077782	2260	https://score.depmap.sanger.ac.uk/gene/SIDG09086
37	FGFR2	ENSG00000066468	2263	https://score.depmap.sanger.ac.uk/gene/SIDG09090
38	FGFR3	ENSG00000068078	2261	https://score.depmap.sanger.ac.uk/gene/SIDG09091
39	FGFR4	ENSG00000160867	2264	https://score.depmap.sanger.ac.uk/gene/SIDG09098
40	FLT4	ENSG00000037280	2324	https://score.depmap.sanger.ac.uk/gene/SIDG09194
41	GART	ENSG00000159131	2618	https://score.depmap.sanger.ac.uk/gene/SIDG09870
42	GGPS1	ENSG00000152904	9453	https://score.depmap.sanger.ac.uk/gene/SIDG10046
43	GSR	ENSG00000104687	2936	https://score.depmap.sanger.ac.uk/gene/SIDG10734
44	HDAC1	ENSG00000116478	3065	https://score.depmap.sanger.ac.uk/gene/SIDG11112
45	HDAC2	ENSG00000196591	3066	https://score.depmap.sanger.ac.uk/gene/SIDG11115
46	HDAC3	ENSG00000171720	8841	https://score.depmap.sanger.ac.uk/gene/SIDG11117
47	HPRT1	ENSG00000165704	3251	https://score.depmap.sanger.ac.uk/gene/SIDG11926
48	IDH1	ENSG00000138413	3417	https://score.depmap.sanger.ac.uk/gene/SIDG12285
49	IFNAR1	ENSG00000142166	3454	https://score.depmap.sanger.ac.uk/gene/SIDG12352
50	IFNAR2	ENSG00000159110	3455	https://score.depmap.sanger.ac.uk/gene/SIDG12353

51	IGF1R	ENSG00000140443	3480	https://score.depmap.sanger.ac.uk/gene/SIDG12412
52	IKBKB	ENSG00000104365	3551	https://score.depmap.sanger.ac.uk/gene/SIDG12924
53	IL2RA	ENSG00000134460	3559	https://score.depmap.sanger.ac.uk/gene/SIDG12945
54	IL3RA	ENSG00000185291	3563	https://score.depmap.sanger.ac.uk/gene/SIDG12949
55	JAK1	ENSG00000162434	3716	https://score.depmap.sanger.ac.uk/gene/SIDG13374
56	JUN	ENSG00000177606	3725	https://score.depmap.sanger.ac.uk/gene/SIDG13419
57	KCNH2	ENSG00000055118	3757	https://score.depmap.sanger.ac.uk/gene/SIDG13511
58	LIG1	ENSG00000105486	3978	https://score.depmap.sanger.ac.uk/gene/SIDG14604
59	LIG3	ENSG00000005156	3980	https://score.depmap.sanger.ac.uk/gene/SIDG14605
60	MAOA	ENSG00000189221	4128	https://score.depmap.sanger.ac.uk/gene/SIDG17179
61	MAP2K1	ENSG00000169032	5604	https://score.depmap.sanger.ac.uk/gene/SIDG17191
62	MAP2K2	ENSG00000126934	5605	https://score.depmap.sanger.ac.uk/gene/SIDG17193
63	MAPK1	ENSG00000100030	5594	https://score.depmap.sanger.ac.uk/gene/SIDG17235
64	MET	ENSG00000105976	4233	https://score.depmap.sanger.ac.uk/gene/SIDG17604
65	MTOR	ENSG00000198793	2475	https://score.depmap.sanger.ac.uk/gene/SIDG20992
66	NFKB1	ENSG00000109320	4790	https://score.depmap.sanger.ac.uk/gene/SIDG21793
67	NTRK3	ENSG00000140538	4916	https://score.depmap.sanger.ac.uk/gene/SIDG22388
68	PARP3	ENSG00000041880	10039	https://score.depmap.sanger.ac.uk/gene/SIDG23934
69	PDGFRA	ENSG00000134853	5156	https://score.depmap.sanger.ac.uk/gene/SIDG24254
70	PDGFRB	ENSG00000113721	5159	https://score.depmap.sanger.ac.uk/gene/SIDG24255
71	PGD	ENSG00000142657	5226	https://score.depmap.sanger.ac.uk/gene/SIDG24457
72	PIK3CA	ENSG00000121879	5290	https://score.depmap.sanger.ac.uk/gene/SIDG24670
73	PIK3CB	ENSG00000051382	5291	https://score.depmap.sanger.ac.uk/gene/SIDG24671
74	PIK3CD	ENSG00000171608	5293	https://score.depmap.sanger.ac.uk/gene/SIDG24672
75	PIK3CG	ENSG00000105851	5294	https://score.depmap.sanger.ac.uk/gene/SIDG24676
76	PNP	ENSG00000198805	4860	https://score.depmap.sanger.ac.uk/gene/SIDG25153
77	POLA1	ENSG00000101868	5422	https://score.depmap.sanger.ac.uk/gene/SIDG25191
78	POLB	ENSG00000070501	5423	https://score.depmap.sanger.ac.uk/gene/SIDG25193
79	POLE	ENSG00000177084	5426	https://score.depmap.sanger.ac.uk/gene/SIDG25201

80	PRKCA	ENSG00000154229	5578	https://score.depmap.sanger.ac.uk/gene/SIDG25787
81	PSMB1	ENSG00000008018	5689	https://score.depmap.sanger.ac.uk/gene/SIDG26071
82	PSMB2	ENSG00000126067	5690	https://score.depmap.sanger.ac.uk/gene/SIDG26072
83	PSMB5	ENSG00000100804	5693	https://score.depmap.sanger.ac.uk/gene/SIDG26077
84	RAF1	ENSG00000132155	5894	https://score.depmap.sanger.ac.uk/gene/SIDG26631
85	RARA	ENSG00000131759	5914	https://score.depmap.sanger.ac.uk/gene/SIDG26706
86	RPL3	ENSG00000100316	6122	https://score.depmap.sanger.ac.uk/gene/SIDG31312
87	RRM1	ENSG00000167325	6240	https://score.depmap.sanger.ac.uk/gene/SIDG33413
88	RXRA	ENSG00000186350	6256	https://score.depmap.sanger.ac.uk/gene/SIDG33553
89	SRC	ENSG00000197122	6714	https://score.depmap.sanger.ac.uk/gene/SIDG36405
90	TLR8	ENSG00000101916	51311	https://score.depmap.sanger.ac.uk/gene/SIDG37785
91	TNFRSF8	ENSG00000120949	943	https://score.depmap.sanger.ac.uk/gene/SIDG38224
92	TOP1	ENSG00000198900	7150	https://score.depmap.sanger.ac.uk/gene/SIDG38338
93	TOP2A	ENSG00000131747	7153	https://score.depmap.sanger.ac.uk/gene/SIDG38342
94	TUBB	ENSG00000196230	203068	https://score.depmap.sanger.ac.uk/gene/SIDG39904
95	TUBD1	ENSG00000108423	51174	https://score.depmap.sanger.ac.uk/gene/SIDG39951
96	TUBE1	ENSG00000074935	51175	https://score.depmap.sanger.ac.uk/gene/SIDG39952
97	TUBG1	ENSG00000131462	7283	https://score.depmap.sanger.ac.uk/gene/SIDG39953
98	TXNRD1	ENSG00000198431	7296	https://score.depmap.sanger.ac.uk/gene/SIDG40025
99	TYMS	ENSG00000176890	7298	https://score.depmap.sanger.ac.uk/gene/SIDG40031
100	YES1	ENSG00000176105	7525	https://score.depmap.sanger.ac.uk/gene/SIDG41320

Supplementary Table S3: Oncology drug targets present in Cancer Dependency Map dataset

S.NO	GENE	ENSEMBL GENE ID	ENTREZ ID	SANGER DEMAP PROJECT SCORE LINK
1	AR	ENSG00000169083	367	https://score.depmap.sanger.ac.uk/gene/SIDG01312
2	ATIC	ENSG00000138363	471	https://score.depmap.sanger.ac.uk/gene/SIDG01760
3	ATOX1	ENSG00000177556	475	https://score.depmap.sanger.ac.uk/gene/SIDG01770
4	BCL2	ENSG00000171791	596	https://score.depmap.sanger.ac.uk/gene/SIDG02177
5	BRAF	ENSG00000157764	673	https://score.depmap.sanger.ac.uk/gene/SIDG02491

6	CCND1	ENSG00000110092	595	https://score.depmap.sanger.ac.uk/gene/SIDG03838
7	CD19	ENSG00000177455	930	https://score.depmap.sanger.ac.uk/gene/SIDG03947
8	CDK4	ENSG00000135446	1019	https://score.depmap.sanger.ac.uk/gene/SIDG04147
9	CHD1	ENSG00000153922	1105	https://score.depmap.sanger.ac.uk/gene/SIDG04542
10	CMPK1	ENSG00000162368	51727	https://score.depmap.sanger.ac.uk/gene/SIDG04945
11	DHFR	ENSG00000178700	1719	https://score.depmap.sanger.ac.uk/gene/SIDG06513
12	EGFR	ENSG00000146648	1956	https://score.depmap.sanger.ac.uk/gene/SIDG07457
13	ERBB2	ENSG00000141736	2064	https://score.depmap.sanger.ac.uk/gene/SIDG07927
14	ESR1	ENSG00000091831	2099	https://score.depmap.sanger.ac.uk/gene/SIDG08129
15	ESR2	ENSG00000140009	2100	https://score.depmap.sanger.ac.uk/gene/SIDG08130
16	FCGR1A	ENSG00000150337	2209	https://score.depmap.sanger.ac.uk/gene/SIDG08944
17	FCGR3A	ENSG00000203747	2214	https://score.depmap.sanger.ac.uk/gene/SIDG08950
18	FDPS	ENSG00000160752	2224	https://score.depmap.sanger.ac.uk/gene/SIDG08977
19	GART	ENSG00000159131	2618	https://score.depmap.sanger.ac.uk/gene/SIDG09870
20	GGPS1	ENSG00000152904	9453	https://score.depmap.sanger.ac.uk/gene/SIDG10046
21	HDAC1	ENSG00000116478	3065	https://score.depmap.sanger.ac.uk/gene/SIDG11112
22	HDAC3	ENSG00000171720	8841	https://score.depmap.sanger.ac.uk/gene/SIDG11117
23	IGF1R	ENSG00000140443	3480	https://score.depmap.sanger.ac.uk/gene/SIDG12412
24	IL2RA	ENSG00000134460	3559	https://score.depmap.sanger.ac.uk/gene/SIDG12945
25	LIG1	ENSG00000105486	3978	https://score.depmap.sanger.ac.uk/gene/SIDG14604
26	LIG3	ENSG00000005156	3980	https://score.depmap.sanger.ac.uk/gene/SIDG14605
27	MTOR	ENSG00000198793	2475	https://score.depmap.sanger.ac.uk/gene/SIDG20992
28	NFKB1	ENSG00000109320	4790	https://score.depmap.sanger.ac.uk/gene/SIDG21793
29	PARP3	ENSG00000041880	10039	https://score.depmap.sanger.ac.uk/gene/SIDG23934
30	PDGFRA	ENSG00000134853	5156	https://score.depmap.sanger.ac.uk/gene/SIDG24254
31	PGD	ENSG00000142657	5226	https://score.depmap.sanger.ac.uk/gene/SIDG24457
32	PIK3CD	ENSG00000171608	5293	https://score.depmap.sanger.ac.uk/gene/SIDG24672
33	POLA1	ENSG00000101868	5422	https://score.depmap.sanger.ac.uk/gene/SIDG25191
34	POLE	ENSG00000177084	5426	https://score.depmap.sanger.ac.uk/gene/SIDG25201

35	PSMB1	ENSG00000008018	5689	https://score.depmap.sanger.ac.uk/gene/SIDG26071
36	PSMB2	ENSG00000126067	5690	https://score.depmap.sanger.ac.uk/gene/SIDG26072
37	PSMB5	ENSG00000100804	5693	https://score.depmap.sanger.ac.uk/gene/SIDG26077
38	RAF1	ENSG00000132155	5894	https://score.depmap.sanger.ac.uk/gene/SIDG26631
39	RPL3	ENSG00000100316	6122	https://score.depmap.sanger.ac.uk/gene/SIDG31312
40	RRM1	ENSG00000167325	6240	https://score.depmap.sanger.ac.uk/gene/SIDG33413
41	TOP1	ENSG00000198900	7150	https://score.depmap.sanger.ac.uk/gene/SIDG38338
42	TOP2A	ENSG00000131747	7153	https://score.depmap.sanger.ac.uk/gene/SIDG38342
43	TUBB	ENSG00000196230	203068	https://score.depmap.sanger.ac.uk/gene/SIDG39904
44	TUBD1	ENSG00000108423	51174	https://score.depmap.sanger.ac.uk/gene/SIDG39951
45	TUBE1	ENSG00000074935	51175	https://score.depmap.sanger.ac.uk/gene/SIDG39952
46	TUBG1	ENSG00000131462	7283	https://score.depmap.sanger.ac.uk/gene/SIDG39953
47	TYMS	ENSG00000176890	7298	https://score.depmap.sanger.ac.uk/gene/SIDG40031

Supplementary Table S4: Fitness genes across 19 cancer-types in the cancer-dependency screen

Gene symbol
BCL2
CCND1
CDK4
CMPK1
EGFR
ERBB2
ESR1
GGPS1
HDAC1
HDAC3
IGF1R
LIG1
MTOR
PGD
TYMS

Supplementary Table S5: List of 15 fitness genes common with 628 priority genes across 19 cancer-types

Gene Symbol
BCL2
CDK4
EGFR
ERBB2
ESR1
HDAC1
HDAC3
IGF1R
MTOR
TYMS

Supplementary Table S6: List of 10 fitness genes common with therapeutic small molecules

Gene symbol
EGFR
ERBB2
IGF1R

Supplementary Table S7: List of 3 fitness genes common with molecules targeted by therapeutic antibodies

S.NO	GENE	ENSEMBL GENE ID	ENTREZ ID	SANGER DEMAP PROJECT SCORE LINK
1	AR	ENSG00000169083	367	https://score.depmap.sanger.ac.uk/gene/SIDG01312
2	ATIC	ENSG00000138363	471	https://score.depmap.sanger.ac.uk/gene/SIDG01760
3	BCL2	ENSG00000171791	596	https://score.depmap.sanger.ac.uk/gene/SIDG02177
4	BRAF	ENSG00000157764	673	https://score.depmap.sanger.ac.uk/gene/SIDG02491
5	CCND1	ENSG00000110092	595	https://score.depmap.sanger.ac.uk/gene/SIDG03838
6	CDK4	ENSG00000135446	1019	https://score.depmap.sanger.ac.uk/gene/SIDG04147
7	CHD1	ENSG00000153922	1105	https://score.depmap.sanger.ac.uk/gene/SIDG04542
8	CMPK1	ENSG00000162368	51727	https://score.depmap.sanger.ac.uk/gene/SIDG04945
9	DHFR	ENSG00000178700	1719	https://score.depmap.sanger.ac.uk/gene/SIDG06513

10	EGFR	ENSG00000146648	1956	https://score.depmap.sanger.ac.uk/gene/SIDG07457
11	ERBB2	ENSG00000141736	2064	https://score.depmap.sanger.ac.uk/gene/SIDG07927
12	FCGR1A	ENSG00000150337	2209	https://score.depmap.sanger.ac.uk/gene/SIDG08944
13	FCGR3A	ENSG00000203747	2214	https://score.depmap.sanger.ac.uk/gene/SIDG08950
14	FDPS	ENSG00000160752	2224	https://score.depmap.sanger.ac.uk/gene/SIDG08977
15	GART	ENSG00000159131	2618	https://score.depmap.sanger.ac.uk/gene/SIDG09870
16	GGPS1	ENSG00000152904	9453	https://score.depmap.sanger.ac.uk/gene/SIDG10046
17	HDAC1	ENSG00000116478	3065	https://score.depmap.sanger.ac.uk/gene/SIDG11112
18	HDAC3	ENSG00000171720	8841	https://score.depmap.sanger.ac.uk/gene/SIDG11117
19	IGF1R	ENSG00000140443	3480	https://score.depmap.sanger.ac.uk/gene/SIDG12412
20	IL2RA	ENSG00000134460	3559	https://score.depmap.sanger.ac.uk/gene/SIDG12945
21	LIG1	ENSG00000105486	3978	https://score.depmap.sanger.ac.uk/gene/SIDG14604
22	LIG3	ENSG00000005156	3980	https://score.depmap.sanger.ac.uk/gene/SIDG14605
23	MTOR	ENSG00000198793	2475	https://score.depmap.sanger.ac.uk/gene/SIDG20992
24	NFKB1	ENSG00000109320	4790	https://score.depmap.sanger.ac.uk/gene/SIDG21793
25	PARP3	ENSG00000041880	10039	https://score.depmap.sanger.ac.uk/gene/SIDG23934
26	PDGFRA	ENSG00000134853	5156	https://score.depmap.sanger.ac.uk/gene/SIDG24254
27	PGD	ENSG00000142657	5226	https://score.depmap.sanger.ac.uk/gene/SIDG24457
28	PIK3CD	ENSG00000171608	5293	https://score.depmap.sanger.ac.uk/gene/SIDG24672
29	POLA1	ENSG00000101868	5422	https://score.depmap.sanger.ac.uk/gene/SIDG25191
30	POLE	ENSG00000177084	5426	https://score.depmap.sanger.ac.uk/gene/SIDG25201
31	PSMB1	ENSG00000008018	5689	https://score.depmap.sanger.ac.uk/gene/SIDG26071
32	PSMB2	ENSG00000126067	5690	https://score.depmap.sanger.ac.uk/gene/SIDG26072
33	PSMB5	ENSG00000100804	5693	https://score.depmap.sanger.ac.uk/gene/SIDG26077
34	RAF1	ENSG00000132155	5894	https://score.depmap.sanger.ac.uk/gene/SIDG26631
35	RPL3	ENSG00000100316	6122	https://score.depmap.sanger.ac.uk/gene/SIDG31312
36	RRM1	ENSG00000167325	6240	https://score.depmap.sanger.ac.uk/gene/SIDG33413
37	TOP1	ENSG00000198900	7150	https://score.depmap.sanger.ac.uk/gene/SIDG38338
38	TOP2A	ENSG00000131747	7153	https://score.depmap.sanger.ac.uk/gene/SIDG38342
39	TUBB	ENSG00000196230	203068	https://score.depmap.sanger.ac.uk/gene/SIDG39904
40	TUBD1	ENSG00000108423	51174	https://score.depmap.sanger.ac.uk/gene/SIDG39951
41	TUBE1	ENSG00000074935	51175	https://score.depmap.sanger.ac.uk/gene/SIDG39952
42	TUBG1	ENSG00000131462	7283	https://score.depmap.sanger.ac.uk/gene/SIDG39953
43	TYMS	ENSG00000176890	7298	https://score.depmap.sanger.ac.uk/gene/SIDG40031

Supplementary Table S8: Cellular targets with excellent fitness effect in cancer-types for which drugs targeting these targets are not approved

References

70. Rody, A.; Karn, T.; Liedtke, C.; Pusztai, L.; Ruckhaeberle, E.; Hanker, L.; Gaetje, R.; Solbach, C.; Ahr, A.; Metzler, D.;et al. A clinically relevant gene signature in triple negative and basal-like breast cancer. *Breast Cancer Res.* **2011**, *13*, R97.
71. Karn, T.; Pusztai, L.; Holtrich, U.; Iwamoto, T.; Shiang, C.Y.; Schmidt, M.; Müller, V.; Solbach, C.; Gaetje, R.; Hanker, L.; et al. Homogeneous Datasets of Triple Negative Breast Cancers Enable the Identification of Novel Prognostic and Predictive Signatures. *PLOS ONE* **2011**, *6*, e28403, doi:10.1371/journal.pone.0028403.
72. Karn, T.; Pusztai, L.; Ruckhaberle, E.; Liedtke, C.; Muller, V.; Schmidt, M.; Metzler, D.; Wang, J.; Coombes, K.R.; Gätje, R.;et al. Melanoma antigen family A identified by the bimodality index defines a subset of triple negative breast cancers as candidates for immune response augmentation. *Eur. J. Cancer* **2012**, *48*, 12–23.
73. Karn, T.; Rody, A.; Muller, V.; Schmidt, M.; Becker, S.; Holtrich, U.; Pusztai, L. Control of dataset bias in combined Affymetrix cohorts of triple negative breast cancer. *Genom. Data* **2014**, *2*, 354–356.
74. Jezequel, P.; Loussouarn, D.; Guerin-Charbonnel, C.; Campion, L.; Vanier, A.; Gouraud, W.; Lasla, H.; Guette, C.; Valo, I.; Verrièle, V.; et al. Gene-expression molecular subtyping of triple-negative breast cancer tumours: importance of immune response. *Breast Cancer Res.* **2015**, *17*, 43.
75. Jezequel, P.; Kerdraon, O.; Hondermarck, H.; Guerin-Charbonnel, C.; Lasla, H.; Gouraud, W.; Canon, J.-L.; Gombos, A.; Dalenc, F.; Delaloge, S.; et al. Identification of three subtypes of triple-negative breast cancer with potential therapeutic implications. *Breast Cancer Res.* **2019**, *21*, 65.
76. Choi, J.; Baldwin, T.M.; Wong, M.; Bolden, J.E.; Fairfax, K.A.; Lucas, E.C.; Cole, R.; Biben, C.; Morgan, C.; Ramsay, K.A.; et al. Haemopedia RNA-seq: a database of gene expression during haematopoiesis in mice and humans. *Nucleic Acids Res.* **2018**, *47*, D780–D785.
77. Rhodes, D.R.; Yu, J.; Shanker, K.; Deshpande, N.; Varambally, R.; Ghosh, D.; Barrette, T.; Pandey, A.; Chinnaiyan, A.M. ONCOMINE: a cancer microarray database and integrated data-mining platform. *Neoplasia* **2004**, *6*, 1–6.
78. Choi, J.; Pacheco, C.M.; Mosbergen, R.; Korn, O.; Chen, T.; Nagpal, I.; Englart, S.; Angel, P.W.; Wells, C.A. Stemformatics: visualize and download curated stem cell data. *Nucleic Acids Res.* **2019**, *47*, D841–D846.
79. Uhlen, M.; Fagerberg, L.; Hallstrom, B.M.; Lindskog, C.; Oksvold, P.; Mardinoglu, A; Sivertsson, Å; Kampf, C; Sjöstedt, E; Asplund A; et al. Proteomics. Tissue-based map of the human proteome. *Science* **2015**, *347*, 1260419.
